# Supplementary material for: Nanoscale grinding: Unlocking the nutrient potential of oxidized phosphate rocks for sustainable fertilizer innovation
Source: PLoS One. 2025 May 9;20(5):e0321095. doi: 10.1371/journal.pone.0321095 (PMC12063831; doi:10.1371/journal.pone.0321095)
Supplement: Attachment — Submitted filename: PONE-D-24-36662.pdf [file pone.0321095.s002.pdf]

PLOS ONE

Nanoscale Grinding: Unlocking the Nutrient Potential of Oxidized Phosphate Rocks for Sustainable Fertilizer Innovation

--Manuscript Draft--

**Manuscript Number:**	PONE-D-24-36662
**Article Type:**	Research Article
**Full Title:**	Nanoscale Grinding: Unlocking the Nutrient Potential of Oxidized Phosphate Rocks for Sustainable Fertilizer Innovation
**Short Title:**	Grinding: Unlocking the Nutrient Potential of Oxidized Phosphate Rocks for Sustainable Fertilizer
**Corresponding Author:**	Houda Khedr<br>New Valley University<br>New Valley, El Kharga, --- Select One --- EGYPT
**Keywords:**	Oxidized phosphate rock; Nanoscale grinding; Acetic acid leaching; High-reactive P fertilizer; Phosphorus dissolution rate; P/Ca liquid fertilizer
**Abstract:**	The current study delves into the transformative effects of intensive grinding to nanoscale upon oxidized phosphate rocks (PRs) of various grades, high (HMP), medium (MMP) and low (LMP) of micro-sizes. Hence, the consequences of these transformative changes on phosphorous dissolution rate of these fractions using acetic acid, was carefully evaluated. The produced high (HNP) and medium (MNP) grades of nano-sized fractions revealed significant changes in their chemical composition, mineralogical, morphological and geometrical properties. Whereas the low grade, LNP, was moderately changed. HNP and MNP exhibited a remarkable increase in amorphicity and Loss on Ignition (LOI) contents (10.62 and 13 wt.%, orderly), surpassing their counterparts (HMP: 6.04 and MMP: 10.92 wt.%). Despite the reduction in their P2O5 contents, HNP (31.23 wt.% and MNP (24.22 wt.%), astoundingly outperformed their micro-sized equivalents (HMP: 35.70 wt.%, MMP: 27.92 wt.%) in P dissolution. Therefore, HNP and MNP emerge as promising high-reactive P fertilizers for direct agricultural use and have a great potential as a source of P/Ca liquid fertilizer after nutrients balancing. So, eco-friendly grinding offers a potential approach to maximize PRs' agronomic potential, but long-term environmental impacts should be evaluated.
**Order of Authors:**	Houda Khedr
	Mohamed O. Ebraheem
	Hussah A. Alshwyeh
	Najla F. Gumaah
	Saedah Rwede AL Mhyawi
	Ahmed H. Ragab
	Ahmed M. Zayed
**Additional Information:**	
**Question**	**Response**
**Financial Disclosure**<br><br>Enter a financial disclosure statement that describes the sources of funding for the work included in this submission. Review the submission guidelines for detailed requirements. View published research articles from *PLOS ONE* for specific	The author(s) received no specific funding for this work.

Powered by Editorial Manager® and ProduXion Manager® from Aries Systems Corporation

examples.<br><br>This statement is required for submission and **will appear in the published article** if the submission is accepted. Please make sure it is accurate.<br><br>**Funded studies**<br>Enter a statement with the following details:<br>• Initials of the authors who received each award<br>• Grant numbers awarded to each author<br>• The full name of each funder<br>• URL of each funder website<br>• Did the sponsors or funders play any role in the study design, data collection and analysis, decision to publish, or preparation of the manuscript?<br><br>Did you receive funding for this work?	
**Competing Interests**<br><br>Use the instructions below to enter a competing interest statement for this submission. On behalf of all authors, disclose any competing interests that could be perceived to bias this work—acknowledging all financial support and any other relevant financial or non-financial competing interests.<br><br>This statement is required for submission and **will appear in the published article** if the submission is accepted. Please make sure it is accurate and that any funding sources listed in your Funding Information later in the submission form are also declared in your Financial Disclosure statement.<br><br>View published research articles from *PLOS ONE* for specific examples.	The authors have declared that no competing interests exist.

Powered by Editorial Manager® and ProduXion Manager® from Aries Systems Corporation

**NO authors have competing interests**<br><br>Enter: *The authors have declared that no competing interests exist.*<br><br>**Authors with competing interests**<br><br>Enter competing interest details beginning with this statement:<br><br>*I have read the journal's policy and the authors of this manuscript have the following competing interests: [insert competing interests here]*<br><br>* typeset	
**Ethics Statement**<br><br>Enter an ethics statement for this submission. This statement is required if the study involved:<br><br>• Human participants<br>• Human specimens or tissue<br>• Vertebrate animals or cephalopods<br>• Vertebrate embryos or tissues<br>• Field research<br><br>Write "N/A" if the submission does not require an ethics statement.<br><br>General guidance is provided below. Consult the submission guidelines for detailed instructions. **Make sure that all information entered here is included in the Methods section of the manuscript.**	Field Research<br>Phosphate Misr company

Powered by Editorial Manager® and ProduXion Manager® from Aries Systems Corporation

**Format for specific study types**<br><br>**Human Subject Research (involving human participants and/or tissue)**<br>• Give the name of the institutional review board or ethics committee that approved the study<br>• Include the approval number and/or a statement indicating approval of this research<br>• Indicate the form of consent obtained (written/oral) or the reason that consent was not obtained (e.g. the data were analyzed anonymously)<br><br>**Animal Research (involving vertebrate animals, embryos or tissues)**<br>• Provide the name of the Institutional Animal Care and Use Committee (IACUC) or other relevant ethics board that reviewed the study protocol, and indicate whether they approved this research or granted a formal waiver of ethical approval<br>• Include an approval number if one was obtained<br>• If the study involved *non-human primates*, add *additional details* about animal welfare and steps taken to ameliorate suffering<br>• If anesthesia, euthanasia, or any kind of animal sacrifice is part of the study, include briefly which substances and/or methods were applied<br><br>**Field Research**<br><br>Include the following details if this study involves the collection of plant, animal, or other materials from a natural setting:<br>• Field permit number<br>• Name of the institution or relevant body that granted permission	
**Data Availability**<br><br>Authors are required to make all data underlying the findings described fully available, without restriction, and from the time of publication. PLOS allows rare exceptions to address legal and ethical concerns. See the PLOS Data Policy and FAQ for detailed information.	Yes - all data are fully available without restriction

A Data Availability Statement describing where the data can be found is required at submission. Your answers to this question constitute the Data Availability Statement and **will be published in the article**, if accepted.<br><br>**Important:** Stating 'data available on request from the author' is not sufficient. If your data are only available upon request, select 'No' for the first question and explain your exceptional situation in the text box.<br><br>Do the authors confirm that all data underlying the findings described in their manuscript are fully available without restriction?	
**Describe where the data may be found in full sentences. If you are copying our sample text, replace any instances of XXX with the appropriate details.**<br><br>• If the data are **held or will be held in a public repository**, include URLs, accession numbers or DOIs. If this information will only be available after acceptance, indicate this by ticking the box below. For example: *All XXX files are available from the XXX database (accession number(s) XXX, XXX.)*.<br>• If the data are all contained **within the manuscript and/or Supporting Information files**, enter the following: *All relevant data are within the manuscript and its Supporting Information files.*<br>• If neither of these applies but you are able to provide **details of access elsewhere**, with or without limitations, please do so. For example:<br><br>*Data cannot be shared publicly because of [XXX]. Data are available from the XXX Institutional Data Access / Ethics Committee (contact via XXX) for researchers who meet the criteria for access to confidential data.*<br><br>*The data underlying the results presented in the study are available from (include the name of the third party*	All relevant data are within the manuscript and its Supporting Information files.

and contact information or URL).<br>• This text is appropriate if the data are owned by a third party and authors do not have permission to share the data.<br><br>* typeset	
Additional data availability information:	

Powered by Editorial Manager® and ProduXion Manager® from Aries Systems Corporation

Click here to access/download;Manuscript;Manuscript..docx

Nanoscale Grinding: Unlocking the Nutrient Potential of Oxidized Phosphate Rocks for Sustainable Fertilizer Innovation

Houda A. Khedr[1*], Mohamed O. Ebraheem[1], Hussah A. Alshwyeh[2], Najla F. Gumaah[3], Saedah Rwede AL-Mhyawi[4], Ahmed H. Ragab[5], Ahmed M. Zayed [6, *]

[1]Geology Department, Faculty of Science, New Valley University, New Valley, Egypt

[2]Biology Department, College of Science, Imam Abdulrahman Bin Faisal University, 1982, Dammam 31441, Saudi Arabia.

[2]Basic & Applied Scientific Research Center (BASRC), Imam Abdulrahman Bin Faisal University, 1982, Dammam 31441, Saudi Arabia.

[3]Chemistry Department, Faculty of Science, Northern Border University, Saudia Arabia.

[4] Chemistry Department, Faculty of Science, University of Jeddah, Jeddah, Saudi Arabia.

[5]Chemistry Department, College of Science, King Khalid University, Abha, 61413, Saudi Arabia

[6]Applied Mineralogy and Water Research Lab (AMWRL), Geology Department, Faculty of Science, Beni-Suef University, Beni Suef, 62521, Egypt

Abstract

The current study delves into the transformative effects of intensive grinding to nanoscale upon oxidized phosphate rocks (PRs) of various grades, high (HMP), medium (MMP) and low (LMP) of micro-sizes. Hence, the consequences of these transformative changes on phosphorous

dissolution rate of these fractions using acetic acid, was carefully evaluated. The produced high (HNP) and medium (MNP) grades of nano-sized fractions revealed significant changes in their chemical composition, mineralogical, morphological and geometrical properties. Whereas the low grade, LNP, was moderately changed. HNP and MNP exhibited a remarkable increase in amorphicity and Loss on Ignition (LOI) contents (10.62 and 13 wt.%, orderly), surpassing their counterparts (HMP: 6.04 and MMP: 10.92 wt.%). Despite the reduction in their P_2O_5 contents, HNP (31.23 wt.% and MNP (24.22 wt.%), astoundingly outperformed their micro-sized equivalents (HMP: 35.70 wt.%, MMP: 27.92 wt.%) in P dissolution. Therefore, HNP and MNP emerge as promising high-reactive P fertilizers for direct agricultural use and have a great potential as a source of P/Ca liquid fertilizer after nutrients balancing. So, eco-friendly grinding offers a potential approach to maximize PRs' agronomic potential, but long-term environmental impacts should be evaluated.

Keywords: Oxidized phosphate rock; Nanoscale grinding; Acetic acid leaching; High-reactive P fertilizer; Phosphorus dissolution rate; P/Ca liquid fertilizer

Corresponding authors:

zayed_2000eg@yahoos.com, ahmed.zayed@science.bsu.edu.eg

houda.mohamed238@gmail.com

1. Introduction

One of the major challenges facing agriculture is the insufficient resource of phosphorus (P) in soils, which is an essential nutrient for enhancing soil fertility and crop production [1]. Phosphate rocks (Rs) are a vital source of all phosphorus (P) industries and play a critical role in

feeding the world's growing population [2]. Additionally, they serve as the primary source of phosphorus-based chemicals [3, 4].

However, a significant portion of soluble P in soil is rapidly converted into forms that reduce its availability to plants [5]. As a result, repeated applications of P compounds may be crucial throughout a crop's life cycle to maintain adequate levels of plant nutrition. Over time, excessive use of soluble P fertilizers can lead to soil saturation and reduced sorption capacity, potentially resulting in increased P losses [6].

The phosphatic ore-based industries have an indirect hazardous environmental impact that can be seen in the increased occurrence of eutrophication. This is correlated with the extreme use of fertilizers for agricultural purposes, which leads to soil and water pollution [7] . The soluble phosphorus that exceeds plant requirements is discharged into aquatic systems, resulting in algal blooms that can block sunlight and cause oxygen depletion, leading to toxicity and mortality of organisms. Consequently, it is crucial to find methods that not only increase soil phosphorus content for agricultural production but also reduce environmental pollution for ecological preservation [8] .

Phosphorites, which are categorized into four types based on their origin: 1) sedimentary marine sediments; 2) igneous deposits ;3) metamorphic deposits; 4) biogenic deposits (bird and bat guano accumulations), are widely distributed in many parts of the world. Most of the phosphate production comes from sedimentary sediments [9].

Egypt is one of the major countries having high reserves of sedimentary phosphate of Late Cretaceous age, with three main districts: Red Sea, Nile Valley and Abu-Tartur deposits. These deposits were formed in a reducing environment and have a grey-to-black colour due to

organic matters enrichment. Over time, geochemical weathering has partially changed its colour to yellowish-brown due to oxidation by the experienced diagenetic processes [10, 11].

Phosphate rocks, PRs, cannot be employed as fertilizer in their pristine form; their low solubility requires prior processing. This is correlated with their associated gangue minerals such as carbonates, silicates, feldspar, mica, carbonate minerals, and clays that require processing [12-14] . These processes include screening, scrubbing, heavy media separation, washing, roasting, calcination, leaching, and flotation [13, 15]. The processing of phosphate ore fractions is significantly influenced by their particle size and had a positive effect on P solubility [16].

To address the ever-growing demands for phosphatic fertilizers, this study aims to develop new types of nonconventional and sustainable fertilizers that could improve fertilizer efficiency and performance through the application of new emerging nano-fertilizers technology. These nano-fertilizers, which can come in powder or liquid forms provide readily available nutrients to plants that can enhance plant production compared to traditional fertilizers.

2. Materials and Methods

2.1. Materials and Chemicals

Oxidized phosphate rocks from the different exposed grades (high, medium, and low) of Duwi Formation at Abu Tartur plateau, were used in the current study. The investigated samples were collected from the eastern to the western part of Liffiyia – Maghrabi sector (25°25′34″N and 30°05′08″E) that located about 50 km west of Kharga Oasis, Western Desert, Egypt. To avoid the malignant impact of inorganic acids (e.g., sulfuric, nitric and hydrochloric acid) although their high leaching capability, organic acetic acid (98%) as one of the most promising leaching agents for calcareous and/or dolomitic gangue materials associated with the phosphatic

deposits was applied in the current study. Also, distilled water (DW) was used as diluting solution.

2.2. Preparation of samples

To prepare the collected oxidized phosphate rocks (PR), the samples of each grade were crushed using a jaw crusher to get a size fraction of < 4.75 mm. Then the crushed samples were delivered to the disc mill to get finer sizes of ≥ 100 µm. The ground samples were mixed carefully till entire homogenization. Then, the produced homogenous mixture was quartered several times to get a representative sample. After washing several times with DW and drying in an oven drier at 70˚C overnight, the sample was smoothly reground in an agate mortar and sieved to get a size < 100 µm. Each one of these ground samples was divided into two halves, one half was packed and labeled for further investigations. Whereas the other one was subjected to intensive grinding several times (sieving & grinding) using a ball mill (RETSCH PM 100/Germany) to get size fractions in nano-size (<100nm). The nano-size fractions were labeled as HNP, MNP and LNP, reflecting the high, medium and low grades of phosphatic deposits, respectively. Whereas those prepared fractions in micro-sizes were labeled as HMP, MMP and LMP, orderly.

2.3. Characterization of the prepared samples

The prepared PR samples in both sizes (micro-and nano-sizes) were characterized using the X-ray fluorescence (XRF) technique (Philips X-ray fluorescence analyzer model PW/2404) to determine their chemical composition, especially the P_2O_5 and CaO contents. Additionally the mineralogical characteristics were examined using both optical microscopy (Nickon Eclipse: LV 100POL) and X-ray diffraction (XRD) techniques, Philips APD-3720 diffractometer (Cu Kα

radiation operated at 20 mA and 40 kV) in the 2θ range of 5°-80° at a scanning speed of 5°/ min. Even though the morphological characteristics and the dominating functional groups on the surface of the addressed samples were investigated using the scanning electron microscopy (SEM) technique (JSM-6700F, JEOL, Tokyo, Japan, beam energy: 20–30 kV, working distance:11.1–12.2 mm) and Fourier transforms infrared (FT-IR) technique (Bruker FTIR-2000 Spectrometer with the mode of reflection at a 4 cm^{-1} resolution), respectively. For SEM analysis, the powder of each sample was installed on stubs and covered with gold using a coating device (JEOL-JSM-420, Japan). Whereas, for the FT-IR study, the sample powder was mixed with KBr (Merck) in a 1:10 ratio to get translucent pellets used for identifying the functional groups of the samples. Furthermore, the Beta surface area (S_{BET}, m^2/ g), total pore volume (V_t, cm^3/ g), and average pore diameter (Dp, nm) of all the PR fractions (HMP, HNP, MMP, MNP, LMP, and LNP) were studied by Surface Area Analyzer (Nova 2000 Quantachrome) in nitrogen adsorption/desorption environment after degassing at 100°C/2h to remove the adsorbed contaminants from their surfaces and pores. The BET surface area (S_{BET}) was estimated by Brunauer-Emmet and Teller model [17], while the total pore volume (Vt) and average pore diameter (Dp) were measured using Barrett-Joyner-Halenda (BJH) formula [18].

2.4. Leaching experiments using acetic acid

To evaluate the solubility of the prepared samples (HMP, HNP, MMP, MNP, LMP, and LNP) of the investigated phosphate deposits by liquified acetic acid, AA (98 %) with specified volume of DW (25 ml), the following experimental parameters were studied at ambient temperature: effect of AA concentration, effect of contact time and regeneration studies. The prevailing experimental conditions of each experimental parameter are compiled in Table (1). After solid-liquid separation using digital centrifuge (Frontier TH5706 /Ohaus/USA) at 8000

rpm/10 min, the liquidous phases were analyzed via inductive coupled plasma optical emission spectrometry (ICP OES/Optima DV) to estimate the dissolved P obtained from the leaching process. For regeneration studies, a 2:1 w/w ratio of AA/PR for each of the prepared samples were used separately and the dissolved P was estimated after centrifuging. Then the solid filtrate fractions were collected and dried overnight at 105 °C to be reused again for another cycle of acid leaching. These fractions were reweighted again before the reconduction of the leaching process to estimate the wasted mass from the previous cycle and to maintain the AA/PA ratio at 2:1 in each new cycle. This step was repeated several times till the approximate consumption of the applied powder of the PR (Table 1).

Table 1. Applied experimental parameters and prevailing conditions during the phosphorous (P) leaching experiments from the prepared phosphatic rocks (PRs) by acetic acid (AA).

**Investigated parameter**	**Prevailing Conditions**					**The other parameters**
**AA/PR ratio (w/w)**	(0.5:1) 0.75: 1.5g	(1:1) 1.5:1.5g	(2:1) 3:1.5g	(3:1) 4.5:1.5g	(4:1) 6:1.5g	25 ml DW, 200 rpm/2h (speed /agitation time).
**Contact time (h)**	½	1	2	4	6	AA/PR (2:1 ratio, 3 g AA: 1.5g PR), 25 ml DW, 200 rpm (speed)
**Regeneration studies of PRs (g)**	Cycle 1 (10:5g)	Cycle 2 (6:3g)	Cycle 3 (4:2g)	Cycle 4 (3:1.5g)	Cycle 5 (1:0.5g)	AA/PR (2:1) ratio, 25 DW, 200 rpm/2h (speed/agitation time).

3. Results and Discussion

3.1 Field characteristics

The phosphatic rocks (PRs) of the current study that were collected from Abu-Tartur plateau, belong to Duwi Formation of Campanian–Maastrichtian age (Fig.1a and b). Such deposits represent one of the largest phosphate reserves in the world, about 70 billion metric tons with different grades up to 30 wt.% P_2O_5 [19]. Also these phosphatic deposits exhibit lateral and vertical facies variation, as well as thickness reduction towards both east and west directions of the sector [20]. The primary or secondary origin of these deposits till now is a matter of debate among scientists [21]. Duwi Formation composed of three dominant members: Abu Tartur Phosphate (lower), Liffiya Shale (middle) and Maghrabi Shale (upper) [20]. The bottom of the lower member is composed mainly of 6 m-thick PR, whereas the top part is up to 5 m-thick of dark grey shale and siltstones intercalations [20]. Such member was considered as the initial marine transgression record for the Late Cretaceous of Egypt that persisted till the deposition of organic-matters/pyrite enriched shale [22, 23]. On the other hand, the middle Liffiya Member is represented by 13 m-thick regressive alternations of light green, medium-grained glauconite-bearing mudstone and siltstones, calcareous siltstone and phosphate. Also, the upper Maghrabi Member (12 m-thick) is composed of a fresh transgression greyish shale with glauconite bed intercalation that is overlain by a shallow marine, regressive, thin phosphorite bed and siliciclastic gravel [21].

3.2. Characterization of prepared samples

The petrographical characteristics of the pristine PRs for the micro-fractions (HMP, MMP& LMP) were examined ~~and photographed~~, using transmitted light optical microscope at different magnifications. The detailed microscopic investigation revealed that the dominant

composition of all the addressed PR grades are mudclasts (peloids) and bioclasts (bone fragments and teeth) [24] with marked privilege of the former in the high-grade PRs compared to the other grades (Figs. 2 and 3). While the non-phosphatic gangue minerals are represented by carbonates (dolomite, calcite & ankerite), quartz, pyrite and evaporites (gypsum and anhydrite), aligning with previous data [25]. Mudclasts are generally presented as rounded to sub-rounded homogeneous pellets embedded in calcareous/ferruginous cement. These pellets are predominantly structureless except for the occasionally occurring ooids that formed by the growth and crystallization of micrometric phosphate layers (Fig. 2e). As well, mudclasts generally display different shade of colors ranging from white to brown in PPL that become isotropic between XPL (Fig. 2a and b), agreeing with other investigations [26] . However, black varieties can be confined to fresh PR samples. On the other hand, phosphatic bioclasts are represented by bone fragments and teeth (Fig.2a, c, and d). Unlike the low-grade PR, bone fragments have special concentration in both high and medium grade PRs. They occur either as angular to subangular prismatic grains or as irregular ones. They are generally anisotropic with low birefringence, grey first-order interference color lamellar twinning and/or undulatory extinction (Fig. 2d). Elongated and prismatic bone fragments occasionally show extinction parallel to their axis of elongation (Fig. 2d) [27, 28]. Sometimes, the core of some of these bone fragments are occupied with disseminated pyrite surrounded by a yellowish oxidized rim [26]. Similarly, the dominant gangue minerals in the high-grade PRs are presented as scattered detrital quartz (Fig.2f), whereas needle-shaped crystals and stellar arrangements of Ca-sulfate (gypsum and anhydrite) are the prevailing gangues in the medium-grade varieties (Fig. 3a). On the other hand, the low-grade PRs are marked by high frequency of dolomite rhombohedra surrounding the phosphatic grains (Fig.3b-c).

The chemical composition of the investigated PRs in both micro- and nano-fractions (HMP, HNP, MMP, MNP, LMP & LNP) were obtained by XRF technique as given in Table (2). The classification of these fractions into high, medium and low-grade PRs is based on their P_2O_5 contents: 35.7, 27. 92 and 8.01 wt. % in HMP, MMP and LMP, respectively (Table 2). Such P_2O_5 contents can be considered as a merit of their phosphatic components (bone-and mud-fragments) as discussed in the petrographical investigations. Likewise, the distinct variations in many of the other major oxides of these fractions were observed, reflecting a drastic difference in their mineralogical composition, particularly their non-phosphatic components. Concerning the PRs micro-fractions, LMP revealed higher contents of Fe_2O_3, SiO_2, MgO, Al_2O_3, MnO and LOI (7.03, 5.96, 9.65, 1.39, 1.22 & 32.27 wt. %, orderly) compared to their counterparts of the other grade fractions: high, HMP, and medium, MMP (Table 2). The high SiO_2 content in LMP can be ascribed to the abundant presence of glauconite and some detrital quartz in this fraction matching with the results of the petrographical investigations. Whereas the approximately high SiO_2 content (4.91 wt.%) in HMP can be correlated to the presence of detrital quartz in this fraction (Fig. 3f). Similarly, the high MgO, MnO and LOI contents in the LMP (Table 2) can be attributed to the intensive presence of ankerite/dolomite and glauconite as was displayed by petrographical investigations.

As well, the remarkable abundance of SO_3 (15.03 wt.%) in the MMP in comparison with the other studied micro-fractions can be correlated to the intensive presence of gypsum/ anhydrite, orderly, on expense of oxidized pyrite via experienced diagenetic processes, aligning with petrographical outcomes and previously reported data [24, 29]. Other oxides (e.g., TiO_2 and Na_2O) occur in all the investigated PR grades in micro-sizes with no distinct variations in agreement with previously reported data [25]. Furthermore, the high depletion of fluorine content

(F= 0.66 wt.%) in the LMP compared to the other comparable grade fractions, not only correlated to its low phosphatic component content but also due to the re-crystallization processes of francolite into brushite [$CaPO_3(OH)\cdot 2H_2O$] that accompanied the experienced long and lasting diagenetic phases [30, 31]. .

Finally, CaO followed the same trend as the P_2O_5 in the studied micro-fractions with respect to the phosphatic components in each of them (Table2).

After intensive grinding to nano-size, it was observed that both Fe_2O_3 and LOI contents in HNP and MNP were enlarged (Table 2). The increase in Fe_2O_3 content could be attributed to the oxidation of the ferrous components of their precursor micro-fractions with grinding [32]. As well, the approximate destruction of the crystalline structure of both phosphatic and non-phosphatic components, not only increased the amorphous nature of HNP and MNP, but also to high susceptibility to adsorb moisture from the surrounding environment [33]. This probably justifies the observable increase in their LOI contents (Table 2). Conversely, the increase in Fe_2O_3 and LOI of the LNP sample was insignificant in comparison with the precursor LMP and the other nano-fractions. This could be attributed to the experienced complexity in the grinding process of its precursor LMP to nano-fraction because of the high LOI content that exceeded 32 wt.% (Table 2). Moreover, the P_2O_5 content was reduced in HNP and MNP, whereas in LNP it was slightly improved from 8.01 to 8.9 wt.% (Table 2). This could also be attributed to the approximate destruction of the francolite in these fractions (HNP & MNP) with higher intensity than that of LNP during the grinding process. Similarly, the CaO and F contents were slightly reduced after grinding, especially in the high and medium grade fractions of nano-size (Table 2). Furthermore, the positive or negative variation of other oxides after grinding was not substantial.

Table 2. Chemical composition of the prepared micro/ nano-sizes high, medium and low grades

Major elements	HMP	HNP	MMP	MNP	LMP	LNP
SiO_2	4.91	6.54	3.09	3.46	5.96	5.47
TiO_2	0.05	0.07	0.04	0.04	0.05	0.06
Al_2O_3	1.10	1.22~~3~~	0.67	0.65	1.39	1.04
Fe_2O_3	3.20	4.79	5.08	5.56	7.03	7.35
MnO	0.24	0.26	0.11	0.12	1.22	0.89
MgO	0.30	0.33	0.18	0.16	9.65	9.64
CaO	41.40	38.43	34.19	33.24	29.48	28.71
Na_2O	0.61	0.53	0.44	0.42	0.18	0.22
K_2O	0.08	0.10	0.20	0.15	0.09	0.08
P_2O_5	35.70	31.23	27.92	24.22	8.01	8.90
SO_3	3.69	3.54	15.03	16.90	3.83	4.27
F	2.18	1.70	1.61	1.51	0.66	0.56
CL	0.07	0.08	0.08	0.08	0.04	0.06
LOI	6.04	10.62	10.92	13.00	32.27	32.64

of phosphate rocks (PRs): HMP, HNP, MMP, MNP, LMP and LNP.

The XRD patterns (Fig.5a-c) of PR samples in both sizes (micro: HMP, MMP & LMP and nano-sizes: HNP, MNP & LNP) revealed that phosphatic and non-phosphatic minerals are the main components but in different ~~intensities~~, matching with the outcomes of petrographical investigations. The phosphatic minerals are represented by francolite (Carbonate-fluorapatite [Ca_5 (PO_4, CO_3)$_3$ (OH, F)]) and brushite (Calcium Phosphate Hydroxide Hydrate [$CaPO_3$ (OH)

$\cdot 2H_2O$]), but with less frequency. The remarkable appearance of these apatite minerals at HMP compared to MMP and LMP elucidates the high P_2O_5 content in such fraction, aligning with XRF data. The minor appearance of brushite mineral in all the PR grades could be attributed to the recrystallization of phosphatic minerals during the experienced diagenetic processes [30]. Non-phosphatic minerals in the HMP are represented by quartz (2θ: 26.01° & 42.47°), pyrite (2θ: 28.23°), and hematite at 2θ: 64° (Fig.4a). Concerning MMP, sulfate minerals such as gypsum (2θ: 29.22°; 31.96° & 48.47°) and anhydrite (2θ: 25.93°) in addition to quartz (2θ: 40.23°) are the main non-phosphatic minerals (Fig.4b). Whereas ankerite (2θ: 24.29°, 31.24°, 41.40°, 43.93°, 45.18°, 51.3°, 59.1°, 60° & 67.67°) is the prevailing non-phosphatic mineral in LMP with appreciable presence of dolomite (2θ: 43.58°), glauconite (2θ: 26.95° & 37.65°), quartz (2θ: 26.17°) and gypsum at 2θ: 33.72° (Fig.4c). The domination of non-phosphatic minerals at the expense of the phosphatic ones explains the low P_2O_5 content of LMP in comparison with the other two grades as assured by the XRF data. After grinding to nano-sized fractions (HNP, MNP, and LNP) a discernible reduction in the intensity of the characterizing peaks of the phosphatic/ non-phosphatic minerals especially at HNP (Fig.4a). On the contrary, the appearance of several surviving peaks of both phosphatic and non-phosphatic minerals with minor shift in their characterizing 2θ, reflects the approximately mild impact of grinding on the crystallinity of LMP (Fig. 4c). Such fragile impact could be attributed to the high LOI content (> 32 wt. %) in the LMP compared to the other investigated grades as displayed by XRF data. This high ratio complicates the grinding process to nano-size.

The FT-IR spectra (Fig.5), displays the main functional groups of both mico- and nano-sizes fractions of the investigated PRs (HMP, HNP, MMP, MNP, LMP and LNP). The broad vibration bands around 3405 and 1630 cm^{-1} in PR micro-fractions (HMP, MMP and LMP), can

be ascribed to the stretching mode of hydroxyl group [34]. Also, the weak absorption bands at 1623-1632 cm^{-1}, were also assigned to the bending mode of water "δ (H_2O)", that are typically associated with hydrous minerals of the investigated samples as glauconite and gypsum, matching with XRD and petrographical data [35]. The larger intensity of hydroxyl groups in both stretching and bending modes in LMP compared to other fractions (HMP & MMP) can be attributed to its significant LOI content and the remarkable presence of hydrous minerals (glauconite & gypsum) in such fraction as elaborated by XRF, XRD and petrographical investigations, respectively. As well, absorption bands at 1428.9, 1428.7 and 1445.6 cm^{-1}, for HMP, MMP & LMP, orderly, can be correlated to the antisymmetrical elastic vibration (ν_3) of carbonate group $(CO_3)^{2-}$ [36] . Whereas the ν_2 elastic vibration of carbonate $(CO_3)^{2-}$ group was shown only in LMP around 879 cm^{-1} [37]. The conspicuous occurrence of $(CO_3)^{2-}$ group in both vibration types in LMP can be correlated to superior attendance of ankerite and dolomite minerals in such fraction compared to HMP and MMP. On the other hand, the antisymmetrical elastic vibration (ν_3) bands at 1045.6, 1046 and 1041.6 cm^{-1} for HMP, MMP and LMP, orderly, can be assigned to the stretching vibrations of Si-O bond of the inorganic components as quartz and glauconite, aligning with XRD and petrographical data, with some preference of HMP on the level of intensity [38]. Additionally, the changing in the vibration bands that reflect the presence of phosphate group $(PO_4)^{3-}$ with ν_4 absorption pattern, was observed at 604.3, 605 & 590 cm^{-1} in HMP, MMP and LMP micro-size PR fractions, orderly [37].

The grinding process to nano-sizes (HNP, MNP & LNP) exerted either positive or negative shifts in the frequencies of the main functional groups of the micro-sizes fractions accompanied by an obvious strengthening in the intensity of some encountered groups such as OH, δ (H_2O), Si-O, $(CO_3)^{2-}$ and $(PO_4)^{3-}$, confirming the successful impact of such process. For

example, the bands that were ascribed to ν_2 and ν_3 elastic vibration of $(CO_3)^{2-}$ group, were remarkably strengthen in LNP although crystallinity reduction of carbonate minerals (ankerite and dolomite). This could be correlated with the probable formation of carbonic acid (H_2CO_3) from the released carbon dioxide (CO_2) and water vapor (H_2O); the release of these volatiles in an approximately closed grinding system has triggered pressure building up, justifying the remerging of these vapors in H_2CO_3 form. The latter probably further dissociate into bicarbonate ions (HCO^{3-}) that could combine with other cations released from the decomposition of other minerals such as potassium (K^+) from glauconite or Ca^{2+} from sulphate/phosphate minerals to form amorphous bicarbonate, explaining the intensification of the absorption bands of $(CO_3)^{2-}$ group in LNP [39]. Similarly, the strength of Si-O and $(PO_4)^{3-}$ groups in the nano-size fractions could be attributed to the gained degree of amorphization that was induced by intensive grinding, especially in the HNP and MNP (Fig.5). Conversely, both modes of water (stretching and bending) were reduced after grinding in LNP (Fig.5). Whereas in HNP and MNP, the absorption bands of these groups were slightly strengthened with grinding [40, 41]. This could be attributed to the enlarged affinity to absorb moisture from the surrounding environment that accompanied the intensive reduction in particle sizes of these fractions, aligning with the LOI contents (Table 2).

The SEM images revealed the particle size distribution and the morphology of PR samples in both sizes (micro: HMP, MMP & LMP and nano-sizes: HNP, MNP & LNP) at different magnifications (Figs. 6- 8). The micro-size fractions (HMP, MMP & LMP) revealed heterogeneity in particle shapes and sizes in accordance with the difference in durability and hardness of their mineral assemblages, especially the LMP, as assured by XRD and petrographical investigations. This heterogeneity could also be attributed to the differences in

the LOI contents among these fractions as approved by XRF data (Table 2). Therefore, the HMP and to a lesser extent MMP revealed larger ratios of tinier particles than their counterpart LMP (Figs.6a-b, 7a-b and 8a-b, orderly). Generally, the common feature in all the addressed micro-size fractions (HMP, MMP & LMP) is the approximate sub-angularity of their particles with occasional appearance of flouc-like structures toward the rim of their agglomerated particles (Figs.6 a-b, 7a-b, and 8a-b, respectively). But with intensive grinding to nano-sizes (HNP, MNP & LNP), these approximately angular particles converted to spheroidal ones through rigorous rubbing against each other, as well as be the action of the grinding balls of various sizes (Figs. 6c-d, 7c-d, and 8c-d, orderly). The spheroidal nature of the produced particles was inversely proportional with the LOI content of their parental materials (HMP, MMP & LMP). In other words, the high LOI ratios contribute to a pervasive complication for the grinding process and hence the progressive agglomeration phenomena for the produced nanoparticles [42, 43] as displayed in Fig. (8c-d). Consequently, the particle-size distribution tends to be uniform at the high-grade nano-fraction (HNP) and to less extent at MNP in comparison with the LNP. This could be taken as a sign for the approximate destruction of the structure of the ~~incorporated~~ minerals (phosphatic and non-phosphatic ones) in these fractions (HMP& MMP) as displayed by the XRD results.

The N_2 adsorption-desorption isotherms of the analyzed PRs (HMP, HNP, MMP, MNP, LMP and LNP) are presented in Fig.(9a-c). These samples revealed type III adsorption-desorption isotherms in correlation with the weak adsorbent-adsorbate interaction [44]. Meanwhile, the displayed hysteresis loops, H_3- type of the IUPAC classification, assure the accommodation of these materials by slit-like pores [44, 45]. Furthermore, these isotherms indicate mono/multi-layers adsorption of nitrogen molecules [46, 47]. As well, all the presented

isotherms, did not exhibit plateau stage (i.e., no equilibrium were attained) at high-pressure (P/P^0 > 0.9), suggesting the presence of open micro-porosity [45, 48]. Moreover, the rapid increase in adsorption branches of these isotherms, could be attributed to the capillary condensation [49, 50]. On the other hand, the intermittent overlap of the isotherm branches at low P/P^0, replicates fragile connectivity among the semi-closed pores of the investigated materials (Fig. 9a-c). But at higher P/P^0, the divergence of these branches, especially in the micro fractions (HMP, MMP and LMP) over the nano-ones (HNP, MNP and LNP), implies the existence of open large pores in juxtaposition to semi-closed ones [51].

In agreement with N_2 isotherm results, the impact of intensive grinding upon the geometrical parameters (S_{BET}, V_t and D_p) of the micro-size PRs, is shown in Table (3). This was elaborated by the remarkable reduction in S_{BET} and V_t of the produced nano-size varieties, especially HNP fraction (17.725 m^2/g & 0.053 c^3/g, respectively). Such reduction could be tied to the demolition of the original crystalline structure of the produced nano- particles as was confirmed by XRD data (Fig. 4). These tiny particles tend to combine and pack together to form larger non-porous agglomerated aggregates by continual compression during the grinding process [52, 53] as shown by SEM images (Figs. 6c-d). This agglomeration phenomena could be interrelated with the liberation of the tightly bounded crystalline water with intensive grinding [54], with noticeable privilege for HNP over MNP and LNP fractions in descending order (Table 2). On the contrary, the limited reduction in S_{BET} (20.79 and V_t of the LNP (20.79 m^2/g & 0.041 c^3/g, respectively) compared to its counterpart fraction (LMP= 29.14 m^2/g & 0.087 c^3/g, orderly), could be attributed to the hydrous nature of the majority of the crystalline phases of the latter (francolite, brushite, gypsum & glauconite) and the domination of carbonate phases (Ankerite & dolomite) as was assured by both XRD and LOI (32.72 wt.%) shown in XRF results (Table 2).

This composition reduced the impact of grinding upon LMP fraction and approximately hampered the complete demolition of the internal structure of the composing phases as was confirmed by XRD data (Fig. 4c).

Table 3. Textural parameters of micro- and nano-size phosphate rock (PR) fractions obtained from N_2 adsorption-desorption isotherms.

**Sample**	**Surface Area (m^2/g) (S_{BET})**	**Total pore volume (cm^3/g) V_t**	**Average pore diameter (nm) D_p**
HMP	42.27	0.13	1.94
HNP	17.73	0.05	1.92
MMP	42.01	0.13	1.93
MNP	26.21	0.07	1.92
LMP	29.14	0.09	1.93
LNP	20.79	0.04	1.91

3.2. Experiments

3.2.1. Effect of Acetic Acid Concentration

The concentration of acetic acid (AA) had a profound influence on phosphorous, ~~P,~~ dissolution from the investigated PR samples (micro: HMP, MMP & LMP and nano-sizes: HNP, MNP & LNP) using variable AA/PR ratios (w/w) that ranged from 0.5:1 to 4:1. To elaborate this influence, the leaching experiments were executed for each sample separately at a constant PR mass (1.5 g), 200 rpm/2h and 25 ml of DW as a liquid medium at room temperature (Fig.10a). For all the investigated samples, it was revealed that the P dissolution rate was progressively

improved with increasing the applied acid ratio from 0.5:1 to 2:1. Beyond this ratio, the improvement in P dissolution rate was irrelevant, indicating equilibrium state attainment [55]. This aligns with the fact that P solubility increase with increasing the medium acidity [56-58]. In such acidic medium, the protonation of the ≡P–O− surface group of the phosphatic minerals (francolite and brushite) by the H^+ protons of the employed acetic acid, act as a sole driving force for P dissolution [59]. However, intervening from the organic ligand (mono-carboxylic group) of the acetic acid upon P dissolution was negligible as it lacks donor atoms (two or more), like in the other organic acids (oxalic and citric acids), capable of forming bi- or multi-dentate mononuclear surface chelates, and that could accelerate cleavage of metal-oxygen bonds to be eventual detached from mineral surfaces [60]. Additionally, it was revealed that the P dissolution rate from both micro- and nano- PR fractions followed the following trend: HNP (622-1994ppm) > MNP (555-1641 ppm) >HMP (173-499 ppm) > MMP (165-468 ppm) >LNP (24-176 ppm) > LMP (3-65 ppm). This trend was not only correlated with the P_2O_5 wt. % contents of these investigated fractions, but also on their particle sizes, and crystallinity/amorphicity nature. But despite the reduction in the P_2O_5 wt. % contents of HNP and MNP, the prevalence of nanoparticles raised the solid/liquid interface, and hence P solubility was improved [16, 61, 62]. Similarly, the gained amorphicity of the phosphatic minerals with intensive grinding in the nano-fractions (specially HNP and MNP) improved their reactivity for acetic acid and hence their P dissolution rate, compared with their crystalline counterparts in the micro-ones, aligning with XRD data. On the contrary, the very low P_2O_5 wt. % content of both LNP and LMP made them occupy such late order, with preference of the former as was demonstrated by XRF data (Table 2). On the light of the current results, the 2:1 ratio of AA/PR was selected for the conduction of the subsequent experiments.

3.2.2. Effect of Reaction Time

The influence of reaction time (0.5, 1, 2, 4 and 6h) on the P dissolution of the investigated HMP, HNP, MMP, MNP, LMP, and LNP samples, employing a fixed AA concentration that achieved AA/PR w/w ratio of 2:1 for each PR sample, was studied (Fig.10b). The prevailing conditions of such leaching experiments are compiled in Table (1). Careful examination of the relationship between the reaction time and P dissolution rate revealed that raising the reaction time above 2h, has an insignificant impact on the P dissolution of both micro/nano size fractions, indicating equilibrium state attainment whatever the available H^+ proton in the solution. Whereas the early stage of the dissolution reaction is highly dependent on the available hydrogen ions of the acetic acid [59, 63]. But with time, the contribution of H^+ protons became weaker at equilibrium. Therefore, 2h was selected as an equilibrium time. Furthermore, the outstanding P dissolution of HNP (1981-2180 ppm) followed by MNP (1375-1477 ppm), signifies the role of both particle size and gained amorphicity of these fractions [61], with some preference of the formers in accordance with its high P_2O_5 content (31.23 wt.%) compared to the latter (27.92 wt.%). On the contrary, the P dissolution of ~~their~~ micro-fractions (HMP and MMP) fluctuate around 400 ppm along the ~~investigated~~ range of reaction time, with some preference of the former (HMP = 380- 431 ppm). This was correlated with particle gain sizes and the crystalline nature of these fractions and their P_2O_5 wt.% contents. Moreover, the coupling among P_2O_5 contents (< 9 wt.%) and the crystallinity nature of both LMP and LNP samples justify their low P dissolution rate that ranged from ≈ 44-50 and 186-198 ppm, orderly.

3.2.3. Regeneration Studies

To assess the potential amount of dissolved P through the leaching processes of the various PRs (HMP, HNP, MMP, MNP, LMP, and LNP), five leaching cycles were executed,

using a fixed AA/PA w/w ratio (2:1) as depicted in Fig. (10c). The other prevailing conditions of conducting these experiments are listed in Table (1). It was revealed that the P dissolution rate of all addressed samples in both micro/nanoscales at the first leaching cycle was reduced compared to the equivalent experiments of the other investigated parameters (acid concentration and reaction time). This was probably assigned to the crowd presence of these fractions' particles within a fixed volume of liquified acetic acid that diminished the solid-liquid interface due to particles agglomeration/stacking, aligning with SEM data (Figs.6-8). Therefore, the hydrogen protons of the acetic acid were hampered from adequate access to the surfaces of the unreacted particles [43]. Furthermore, the progressive decline in P dissolution was observed with each applied leaching cycle for all the investigated samples (Fig. 10). But P dissolution continuity till the 5th leaching cycle with a remarkable performance of HNP (1495-307 ppm) and MNP (1088-253 ppm) over the other samples, validates the intervening roles of both nano scale of particles/amorphicity upon the P solubility during the successive leaching cycles [52, 64].

3.2.4. Other leached Nutrients Besides Phosphorus

Phosphate rock as a natural fertilizer, is a source of many macro- and micronutrients besides phosphorus [65]. The macronutrients P, K, Ca, Mg, and S are important for various metabolic processes as protein synthesis, chlorophyll formulation and enzyme production that are essential for plant growth [66]. They also serve as plant defense against insect attacks [66]. Whereas the micronutrients like Fe, Si, Al, Zn, and Mn, are crucial for numerous processes such as CO_2 fixation, synthesis of enzymes and proteins, N fixation and metabolism, as well as plant disease prevention although their absorption in trace amounts [67]. However, excessive levels of these micronutrients can be toxic. So, to evaluate the efficiency of the applied acid leaching process on the solubility of other nutrients besides phosphorus, a fixed AA/PR ratio of 2:1 (3:1.5

g, w/w), using 2h/200 rpm and 25 ml DW at ambient temperature, was employed for each PR sample. Like P, the concentration of these nutrients in the separated liquid phases after an adequate centrifuging process, were determined using ICP OES (Table 4). The nano- PR fractions (HNP, MNP and LNP) revealed enhanced dissolution rate of the investigated macro and-micronutrient than micro- fractions (HMP, MMP and LMP). These results nominate the investigated samples, especially HNP and MNP in descending order, to be utilized as a high-reactive P fertilizer for direct agricultural applications. Where the slow P release can provide a constant supply to the plant along the entire growth period, leading to a better plant growth and higher biomass yield and hence more P uptake [68]. This algin with the fact that nanoparticles of PRs can be absorbed onto the clays of the soil, hampering their fixation and facilitating their release into the soil solution that can be utilized by the plant [69]. This demonstrates the commercial viability of such deposits as an alternative to chemical fertilizer. Similarly, a liquid fertilizer can be produced grounding on the leached liquid phase from both HNP and MNP fractions that contains significant amounts of phosphorus (1991.4 & 1638.2 ppm, orderly) and calcium (7331.4 & 4329.3 ppm, orderly), which are valuable nutrients for plants. However, the other nutrient concentrations may need adjustment to create a well-balanced and effective liquid fertilizer. For example, the relatively low potassium concentration from both fractions signifies that potassium may be required to make these liquid phases a well-balanced fertilizer. As well, the leached liquidous phases of LNP can be applied as Ca/Mg/S rich liquid fertilizer after conducting the appropriate balancing procedures for some of their low nutrients, with some preference of the latter fraction. Therefore, further analyses and testing are required to optimize the nutrient ratios, levels of toxic heavy metals (Cr, As, Cd, Pb, U, V & Hg) and to ensure that the produced liquid fertilizer meets the specific requirements of the target plants and soil

conditions, but this is out of the scope of the current study and will be investigated in a sperate future study.

Table 4. Chemical composition of some selected nutrients in the liquidous phase after the applied acetic acid (AA) leaching process for the prepared phosphatic rocks (PRs).

**Component (ppm)**	**PRs**					
	**HMP**	**HNP**	**MMP**	**MNP**	**LMP**	**LNP**
**Phosphorus (P)**	492.5	1991.4	464.5	1638.2	61.6	173.1
**Calcium (Ca)**	1643.1	7331.4	1190.5	4329.3	5769.4	7781.7
**Sulfur (S)**	168.1	179	144.3	263.1	184.9	364.5
**Magnesium (Mg)**	56.7	101.5	39.3	66	2554.9	2823.4
**Potassium (K)**	13.1	39.9	11.9	58.9	25.7	33.4
**Sodium (Na)**	56.8	173.2	50.2	134.2	30.1	61
**Aluminum (Al)**	2.1	2.6	3.4	4.5	33	17.2
**Iron (Fe)**	0.2	0.2	1.4	0.8	63.8	2.3
**Silicon (Si)**	62.4	848.2	25.7	370.3	119.5	677.2

4. Conclusion

The outcomes of the present work can be drawn as follow:

- ✓ The impact of intensive grinding to nanoscale on the chemical composition, mineralogical, morphological and geometrical characteristics of different grades of ~~oxidized~~ PRs (high, medium and low-grade), was carefully investigated.
- ✓ Unlike LNP, the intensive grinding increased the amorphicity of HNP and MNP and raised their LOI contents (10.62 and 13 wt.%, orderly) in a surprising manner compared to their equivalent HMP and MMP (6.04 and 10.92 wt.%, respectively).

- ✓ The grinding process to nanoscale reduced the S_{BET} and the V_t of the HNP, MNP and LNP as a consequence of agglomeration phenomena that was assigned to their high LOI contents compared with their micro-scale counterparts (HMP, MMP and LMP).
- ✓ The P dissolution rate of the precursor micro-scale fractions and their modified derivatives all over the applied experimental parameters traced the following trend: HNP> HNP> HMP > MMP> LNP> LMP.
- ✓ Despite the noticeable P_2O_5 wt.% reduction of HNP and MNP with intensive grinding, their remarkable P dissolution rates (≈ 620 to 2180 ppm and ≈ 550-1640 ppm, orderly) compared to their counterpart micro-fractions, HM (≈ 173 to 499 ppm) and MMP (≈ 165 to 468 ppm), can be tied with their gained amorphicity and their nanoscale particles.
- ✓ The P dissolution rate of all investigated PRs at the first leaching cycle of regeneration studies was obviously reduced compared to equivalent leaching experiments of other investigated parameters (acid concentration and contact time), as a reflection of the profound role of particles agglomeration within the acetic acid solution that hampered H^+ from adequate access to the surfaces of the unreacted particles.
- ✓ The P dissolution continuity till the 5th leaching cycle with preference of HNP and MNP over the others, aligns with the fact of particle size reduction during the successive leaching cycles.
- ✓ HNP and MNP can be considered as a promising source of high-reactive P fertilizer for direct agricultural applications instead of chemical fertilizer. However, long-term effects of nano-sized particles on soil properties, nutrient dynamics, and potential ecological consequences should be evaluated to ensure sustainable agricultural practices.

- ✓ HNP and MNP can provide potential candidates for use as P/Ca rich liquid fertilizers with adequate macro and-micronutrients, whereas LNP can be counted as a source of Ca/Mg/S rich liquid fertilizer after conducting the appropriate balancing procedures for some of their low nutrients.
- ✓ Finally, to maximize the agronomic potential of widely distributed oxidized phosphate deposits, intensive mechanical grinding can be adapted as an eco-friendly protocol.

References

1. Boateng A, Owusu-Bennoah E. ASSESSMENT OF PHOSPHORUS AVAILABILITY FROM UNREACTIVE TOGO PHOSPHATE ROCK BY LEGUME AND CEREAL CROPS IN TWO GHANAIAN SOILS. International Journal of Agriculture. 2020;5(1):1-24.

2. Nancharaiah YV, Mohan SV, Lens PNL. Chapter 4.5 - Removal and Recovery of Metals and Nutrients From Wastewater Using Bioelectrochemical Systems. In: Mohan SV, Varjani S, Pandey A, editors. Microbial Electrochemical Technology: Elsevier; 2019. p. 693-720.

3. Özer AK. The characteristics of phosphate rock for upgrading in a fluidized bed. Advanced Powder Technology. 2003;14(1):33-42. doi: https://doi.org/10.1163/156855203762469885.

4. Dar SA, Khan K, Birch W. Sedimentary: phosphates. Reference Module in Earth Systems and Environmental Sciences. 2017:1-17.

5. Johan PD, Ahmed OH, Omar L, Hasbullah NA. Phosphorus Transformation in Soils Following Co-Application of Charcoal and Wood Ash2021; 11(10).

6. Xiong L, Wang P, Kopittke PM. Tailoring hydroxyapatite nanoparticles to increase their efficiency as phosphorus fertilisers in soils. Geoderma. 2018;323:116-25. doi: https://doi.org/10.1016/j.geoderma.2018.03.002.

7. Khan MN, Mohammad F. Eutrophication: challenges and solutions. Eutrophication: Causes, Consequences and Control: Volume 2. 2014:1-15.

8. Alewell C, Ringeval B, Ballabio C, Robinson DA, Panagos P, Borrelli P. Global phosphorus shortage will be aggravated by soil erosion. Nature Communications. 2020;11(1):4546. doi: https://doi.org/10.1038/s41467-020-18326-7.

9. De Boer MA, Wolzak L, Slootweg JC. Phosphorus: reserves, production, and applications. Phosphorus recovery and recycling. 2019:75-100.

10. Filippelli GM. Phosphate rock formation and marine phosphorus geochemistry: The deep time perspective. Chemosphere. 2011;84(6):759-66. doi: https://doi.org/10.1016/j.chemosphere.2011.02.019.

11. El-Dahhar MA. Diagnetic pyrite framboids in the phosphate deposits of Abu-Tartur area, Western Desert, Egypt. Journal of African Earth Sciences (1983). 1987;6(6):807-11. doi: https://doi.org/10.1016/0899-5362(87)90037-6.

12. Ruan Y, He D, Chi R. Review on Beneficiation Techniques and Reagents Used for Phosphate Ores. Minerals. 2019;9(4):253. doi: https://doi.org/10.3390/min9040253. PubMed PMID: doi:10.3390/min9040253.

13. Gharabaghi M, Noaparast M, Irannajad M. Selective leaching kinetics of low-grade calcareous phosphate ore in acetic acid. Hydrometallurgy. 2009;95(3):341-5. doi: https://doi.org/10.1016/j.hydromet.2008.04.011.

14. Zapata F, Roy RN. Use of phosphate rocks for sustainable agriculture. FAO Fertilizer and Plant Nutrition Bulletin. 2004:1-148.

15. Ghorbani F, Kamari S, Zamani S, Akbari S, Salehi M. Optimization and modeling of aqueous Cr(VI) adsorption onto activated carbon prepared from sugar beet bagasse agricultural waste by application of response surface methodology. Surfaces and Interfaces. 2020;18:100444. doi: https://doi.org/10.1016/j.surfin.2020.100444.

16. Gharabaghi M, Irannajad M, Noaparast M. A review of the beneficiation of calcareous phosphate ores using organic acid leaching. Hydrometallurgy. 2010;103(1):96-107. doi: https://doi.org/10.1016/j.hydromet.2010.03.002.

17. Brunauer S, Emmett PH, Teller E. Adsorption of gases in multimolecular layers. Journal of the American chemical society. 1938;60(2):309-19.

18. Barrett EP, Joyner LG, Halenda PP. The determination of pore volume and area distributions in porous substances. I. Computations from nitrogen isotherms. Journal of the American Chemical society. 1951;73(1):373-80.

19. Glenn CR, Arthur MA. Anatomy and origin of a Cretaceous phosphorite-greensand giant, Egypt. Sedimentology. 1990;37(1):123-54. doi: https://doi.org/10.1111/j.1365-3091.1990.tb01986.x.

20. Banerjee S, Farouk S, Nagm E, Choudhury TR, Meena SS. High Mg-glauconite in the Campanian Duwi Formation of Abu Tartur Plateau, Egypt and its implications. Journal of African Earth Sciences. 2019;156:12-25. doi: https://doi.org/10.1016/j.jafrearsci.2019.05.001.

21. Ahmad F, Farouk S, Abd El-Moghny MW. A regional stratigraphic correlation for the upper Campanian phosphorites and associated rocks in Egypt and Jordan. Proceedings of the Geologists' Association. 2014;125(4):419-31. doi: https://doi.org/10.1016/j.pgeola.2014.06.002.

22. El Nady MM, Hammad MM. Organic richness, kerogen types and maturity in the shales of the Dakhla and Duwi formations in Abu Tartur area, Western Desert, Egypt: Implication of Rock–Eval pyrolysis. Egyptian Journal of Petroleum. 2015;24(4):423-8. doi: https://doi.org/10.1016/j.ejpe.2015.10.003.

23. El-Kammar A. Oil shale resources in Egypt: the present status and future vision. Arabian Journal of Geosciences. 2017;10(19):439. doi: https://doi.org/10.1007/s12517-017-3152-4.

24. Baioumy H, Tada R. Origin of Late Cretaceous phosphorites in Egypt. Cretaceous Research. 2005;26(2):261-75. doi: https://doi.org/10.1016/j.cretres.2004.12.004.

25. Awadalla GS. Geochemistry and microprobe investigations of Abu Tartur REE-bearing phosphorite, Western Desert, Egypt. Journal of African Earth Sciences. 2010;57(5):431-43. doi: https://doi.org/10.1016/j.jafrearsci.2009.11.009.

26. Baioumy HM. Iron–phosphorus relationship in the iron and phosphorite ores of Egypt. Geochemistry. 2007;67(3):229-39. doi: https://doi.org/10.1016/j.chemer.2004.10.002.

27. Baioumy H, Tada R, Gharaie M. Geochemistry of Late Cretaceous phosphorites in Egypt: implication for their genesis and diagenesis. Journal of African Earth Sciences. 2007;49(1-2):12-28.

28. Salama W, El-Kammar A, Saunders M, Morsy R, Kong C. Microbial pathways and palaeoenvironmental conditions involved in the formation of phosphorite grains, Safaga District, Egypt. Sedimentary Geology. 2015;325:41-58. doi: https://doi.org/10.1016/j.sedgeo.2015.05.004.

29. Baioumy HM. Effect of the depositional environment on the compositional variations among the phosphorite deposits in Egypt. Russian Geology and Geophysics. 2013;54(4):454-63. doi: https://doi.org/10.1016/j.rgg.2013.03.007.

30. El-Badry M, Elbarbary T, Ibrahim I, Abdel-Fatah Y. Azotobacter vinelandii evaluation and optimization of Abu Tartur Egyptian phosphate ore dissolution. Saudi J Pathol Microbiol. 2016;1:80-93. doi: https://doi.org/10.21276/sjpm.2016.1.3.2.

31. Abou El-Anwar EA, Mekky HS, Abd El Rahim SH, Aita SK. Mineralogical, geochemical characteristics and origin of Late Cretaceous phosphorite in Duwi Formation (Geble Duwi Mine), Red Sea region, Egypt. Egyptian Journal of Petroleum. 2017;26(1):157-69. doi: https://doi.org/10.1016/j.ejpe.2016.01.004.

32. Kuai J, Zhang H. Research on generation and polishing mechanisms of nano grain α-Fe2O3 in precision electrolytic in process dressing (ELID) grinding. Procedia Manufacturing. 2019;37:425-30.

33. Tole I, Habermehl-Cwirzen K, Cwirzen A. Mechanochemical activation of natural clay minerals: an alternative to produce sustainable cementitious binders – review. Mineralogy and Petrology. 2019;113(4):449-62. doi: https://doi.org/10.1007/s00710-019-00666-y.

34. Guillaume N, Idriss YM, Ech-Chahad A. Transformation of Phosphorite Into Nano-Phosphate in Alkaline Medium.

35. Zayed AM, Metwally BS, Masoud MA, Mubarak MF, Shendy H, Petrounias P, et al. Facile synthesis of eco-friendly activated carbon from leaves of sugar beet waste as a superior nonconventional adsorbent for anionic and cationic dyes from aqueous solutions. Arabian Journal of Chemistry. 2023;16(8):104900. doi: https://doi.org/10.1016/j.arabjc.2023.104900.

36. Tagaev I, Doniyarov N, Asrorov A, Murodov I, Usanbaev NK, Temirov U. Distinctive IR-Spectroscopic Features of Functional Groups of Low-Grade Phosphorites After Microbiological and Acid Processing. Land Science. 2020;2(1):p43-p. doi: https://doi.org/10.30560/ls.v2n1p43.

37. Mao X, Lu Q, Mo W, Xin X, Chen X, He Z. Phosphorus availability and release pattern from activated dolomite phosphate rock in Central Florida. Journal of agricultural and food chemistry. 2017;65(23):4589-96. doi: https://doi.org/10.1021/acs.jafc.7b01037.

38. Parolo ME, Savini MC, Loewy RM. Characterization of soil organic matter by FT-IR spectroscopy and its relationship with chlorpyrifos sorption. Journal of Environmental Management. 2017;196:316-22. doi: https://doi.org/10.1016/j.jenvman.2017.03.018.

39. Toan S, O'Dell W, Russell CK, Zhao S, Lai Q, Song H, et al. Thermodynamics of NaHCO3 decomposition during Na2CO3-based CO2 capture. Journal of Environmental Sciences. 2019;78:74-80. doi: https://doi.org/10.1016/j.jes.2018.07.005.

40. Arnold R, Terfort A, Wöll C. Determination of molecular orientation in self-assembled monolayers using IR absorption intensities: The importance of grinding effects. Langmuir. 2001;17(16):4980-9. doi: https://doi.org/10.1021/la010202o.

41. Cheng Y-h, Tong B-q, Sun X-h, Zhang J-y. On the grinding effects of high-silicon iron tailings. Environmental Science and Pollution Research. 2022;29(48):73326-40. doi: http://Doi.org/10.1007/s11356-022-20964-x.

42. Mofrad RN, Sadrnezhaad SK, Khaki JV. Mechanism of nanostructured fluorapatite formation from CaO, CaF2 and P2O5 precursors by mechanochemical synthesis. Progress in Reaction Kinetics and Mechanism. 2018;43(3-4):201-10. doi: https://doi.org/10.3184/146867818X15233705894419.

43. Zhang X-m, Li Y, Hu C, He Z-q, Wen M-x, Gai G-s, et al. Enhanced Phosphorus Release from Phosphate Rock Activated with Lignite by Mechanical Microcrystallization: Effects of Several Typical Grinding Parameters. Sustainability. 2019;11(4):1068. doi: https://doi.org/10.3390/su11041068. PubMed PMID: doi:10.3390/su11041068.

44. Thommes M, Kaneko K, Neimark AV, Olivier JP, Rodriguez-Reinoso F, Rouquerol J, et al. Physisorption of gases, with special reference to the evaluation of surface area and pore size distribution (IUPAC Technical Report). Pure and Applied Chemistry. 2015;87(9-10):1051-69. doi: https://doi.org/10.1515/pac-2014-1117.

45. Boycheva S, Zgureva D. Surface studies of fly ash zeolites via adsorption/desorption isotherms. Bulg Chem Commun. 2016;48:101-7.

46. Ahmed DS, El-Hiti GA, Yousif E, Hameed AS, Abdalla M. New eco-friendly phosphorus organic polymers as gas storage media. Polymers. 2017;9(8):336. doi: https://doi.org/10.3390/polym9080336.

47. Hanyabui E, Obeng Apori S, Agyei Frimpong K, Atiah K, Abindaw T, Ali M, et al. Phosphorus sorption in tropical soils. 2020.

48. Saoiabi S, El Asri S, Laghzizil A, Coradin T, Lahlil K. Nanoporous surface of organofunctionalized hydroxyapatite fabricated from natural phosphate rock. Materials Letters. 2010;64(24):2679-81. doi: https://doi.org/10.1016/j.matlet.2010.09.013.

49. Abubakar M, Tamin MN, Saleh MA, Uday MB, Ahmad N. Preparation and characterization of a nigerian mesoporous clay-based membrane for uranium removal from underground water. Ceramics International. 2016;42(7):8212-20. doi: https://doi.org/10.1016/j.ceramint.2016.02.031.

50. Abubakar M, Mohd Noor SFb, Ahmad N. Effect of milling time on the performance of ceramic membrane from ball clay for the treatment of nickel plating wastewater. Journal of the Australian Ceramic Society. 2019;55(3):667-79. doi: https://doi.org/10.1007/s41779-018-0276-2.

51. Mastalerz M, He L, Melnichenko YB, Rupp JA. Porosity of Coal and Shale: Insights from Gas Adsorption and SANS/USANS Techniques. Energy Fuels. 2012;26(8):5109-20. doi: https://doi.org/10.1021/ef300735t.

52. Lim HH, Gilkes RJ, McCormick PG. Beneficiation of rock phosphate fertilisers by mechano-milling. Nutrient Cycling in Agroecosystems. 2003;67(2):177-86. doi: https://doi.org/10.1023/A:1025505315247.

53. Giroto A, Fidélis S, Ribeiro C. Controlled release from hydroxyapatite nanoparticles incorporated into biodegradable, soluble host matrixes. RSC advances. 2015;5(126):104179-86. doi: http://doi.ogr.10.1039/C5RA17669G.

54. Widayat W, Hadiyanto H, Wardani PW, Az Zuhra U, Prameswari J. Preparation of KI/Hydroxyapatite Catalyst from Phosphate Rocks and Its Application for Improvement of Biodiesel Production. Molecules [Internet]. 2020; 25(11).

55. Zayed AM, Abdel Wahed MSM, Mohamed EA, Sillanpää M. Insights on the role of organic matters of some Egyptian clays in methyl orange adsorption: Isotherm and kinetic studies. Applied Clay Science. 2018;166:49-60. doi: https://doi.org/10.1016/j.clay.2018.09.013.

56. Doolette AL, Smernik RJ. Soil organic phosphorus speciation using spectroscopic techniques. Phosphorus in action: Biological processes in soil phosphorus cycling. 2011:3-36.

57. Moreno Quevedo AP, Osorio Vega NW, González Murillo OA. In vitro dissolution of acidulated rock phosphate by phosphate solubilizing microorganisms. Acta Biológica Colombiana. 2015;20(2):65-71. doi: http://dx.doi.org/10.15446/abc.v20n2.42713

58. Tõnsuaadu K, Kallas J, Kallaste T, Urtson K, Einard M, Martin R, et al. Estonian Phosphate Rock Dissolution in Hydrochloric Acid: Optimization of Acid Dosage and Concentration. Minerals [Internet]. 2023; 13(4).

59. Wang D, Xie Y, Jaisi DP, Jin Y. Effects of low-molecular-weight organic acids on the dissolution of hydroxyapatite nanoparticles. Environmental Science: Nano. 2016;3(4):768-79. doi: https://doi.org/10.1039/C6EN00085A.

60. Basak BB. Phosphorus Release by Low Molecular Weight Organic Acids from Low-Grade Indian Rock Phosphate. Waste and Biomass Valorization. 2019;10(11):3225-33. doi: https://doi.org/10.1007/s12649-018-0361-3.

61. Sınırkaya M, Özer AK, Gulaboglu MS. Kinetics of dissolution of Mardin-Mazıdaği (Turkey) phosphate ore in dilute phosphoric acid solutions. Mining, Metallurgy & Exploration. 2010;27(2):110-5. doi: https://doi.org/10.1007/BF03402387.

62. Sınırkaya M, Özer AK, Gülaboglu MS. Investigation of the changes of P2O5 content of phosphate rock during simultaneous calcination/sulfation. Powder Technology. 2011;211(1):72-6. doi: https://doi.org/10.1016/j.powtec.2011.03.036.

63. Guidry MW, Mackenzie FT. Experimental study of igneous and sedimentary apatite dissolution: Control of pH, distance from equilibrium, and temperature on dissolution rates. Geochimica et Cosmochimica Acta. 2003;67(16):2949-63. doi: https://doi.org/10.1016/S0016-7037(03)00265-5.

64. Kaptay G. On the size and shape dependence of the solubility of nano-particles in solutions. International Journal of Pharmaceutics. 2012;430(1):253-7. doi: https://doi.org/10.1016/j.ijpharm.2012.03.038.

65. Abdel-Hakeem M, El-Habaak G. The potential production of rock-based fertilizer and soil conditioner from phosphate mine wastes: A case study from Abu-Tartur plateau in the Western Desert of Egypt. Journal of Cleaner Production. 2021;329:129761. doi: https://doi.org/10.1016/j.jclepro.2021.129761.

66. Tripathi DK, Singh S, Gaur S, Singh S, Yadav V, Liu S, et al. Acquisition and homeostasis of iron in higher plants and their probable role in abiotic stress tolerance. Frontiers in Environmental Science. 2018;5:86. doi: https://doi.org/10.3389/fenvs.2017.00086.

67. Barker AV, Pilbeam DJ. Handbook of plant nutrition: CRC press; 2015.

68. Adhikari T, Kundu S, Meena V, Rao AS. Utilization of nano rock phosphate by maize (Zea mays L.) crop in a vertisol of Central India. Journal of Agricultural Science and Technology A. 2014;4(5A).

69. Adhikary S. Nano-fertilizers and its balanced application for plant Nutrition.

Figure

Click here to access/download;Figure;Figure.pptx

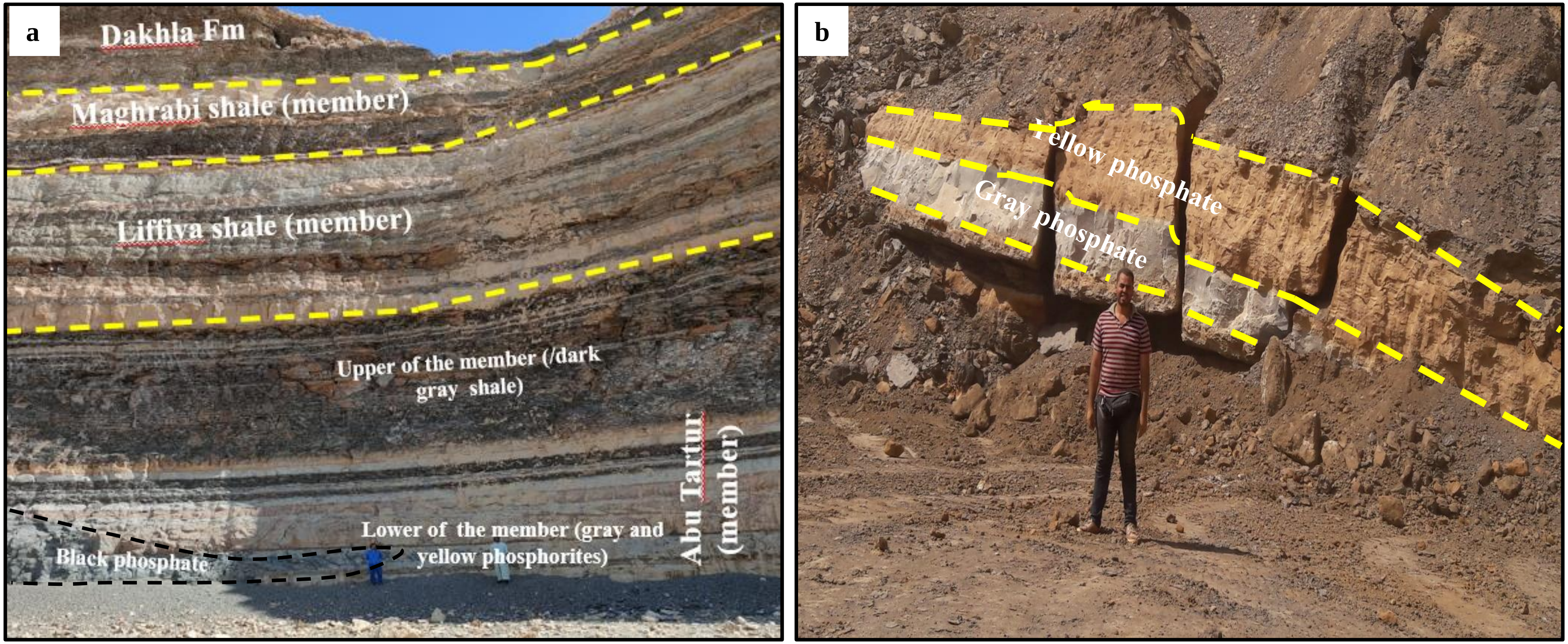

Fig. 1

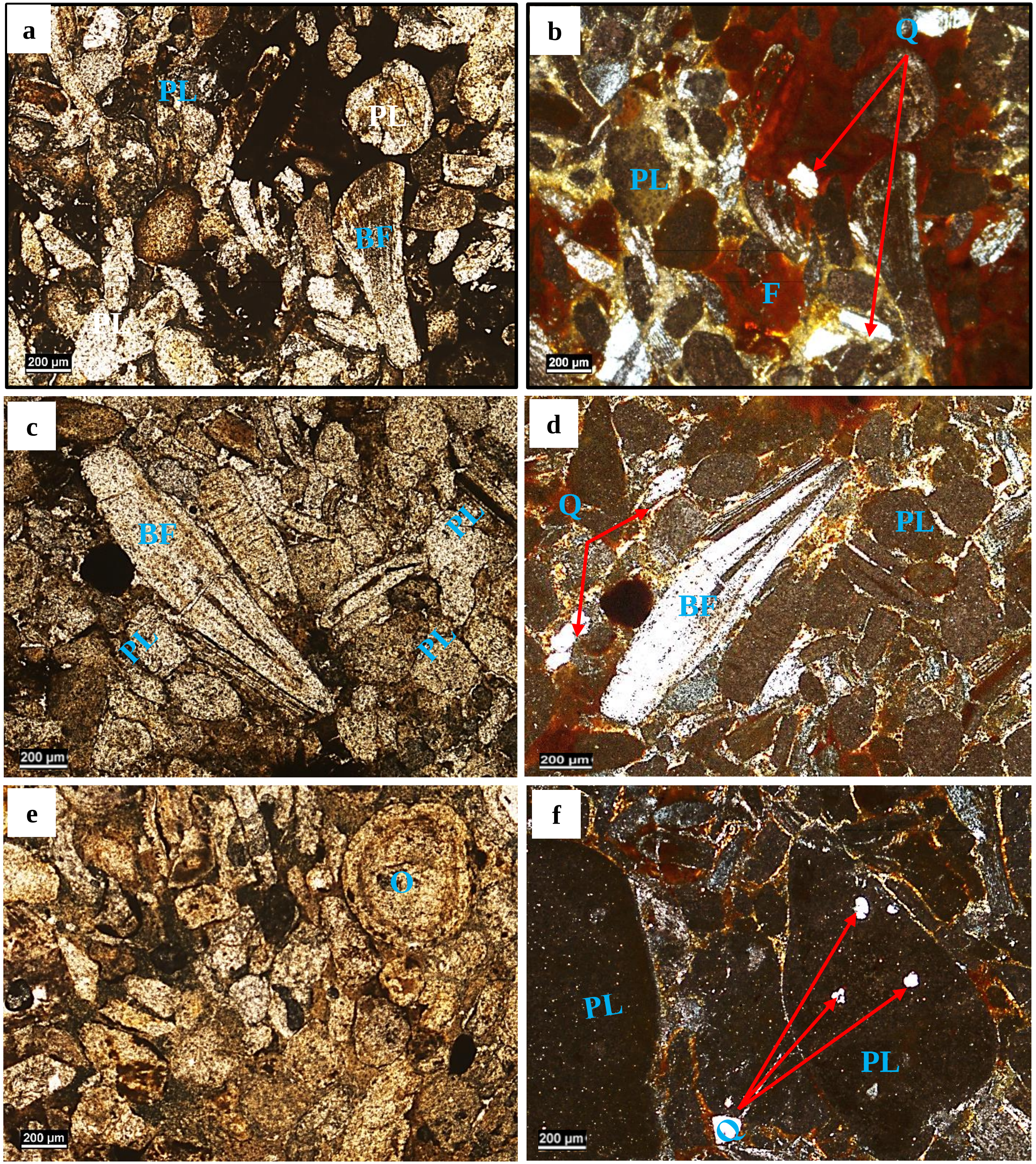
a
PL
PL
BF
200 µm
b
Q
PL
F
200 µm
c
BF
PL
PL
PL
200 µm
d
Q
PL
BF
200 µm
e
O
200 µm
f
PL
PL
Q
200 µm

Fig. 2

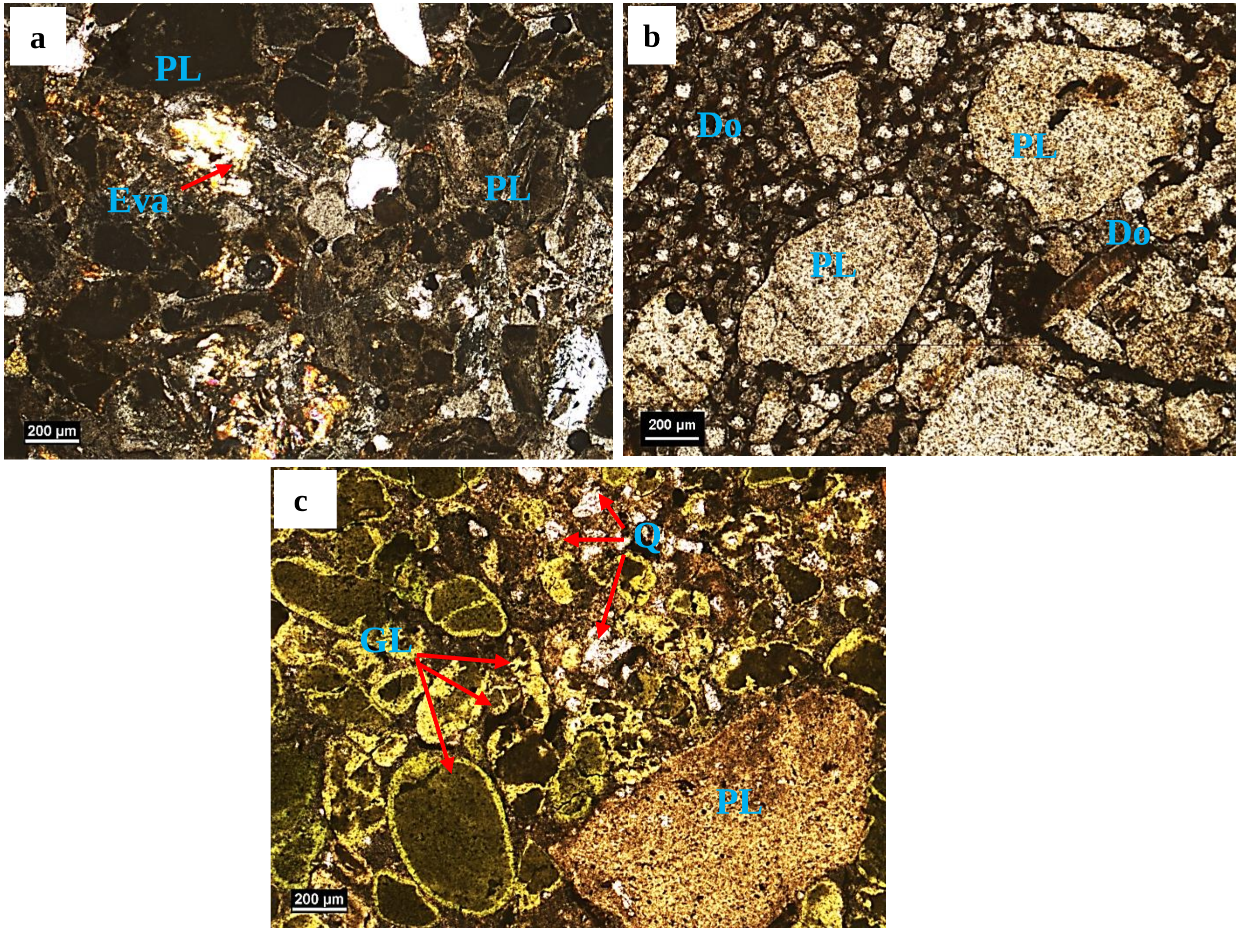

Fig. 3

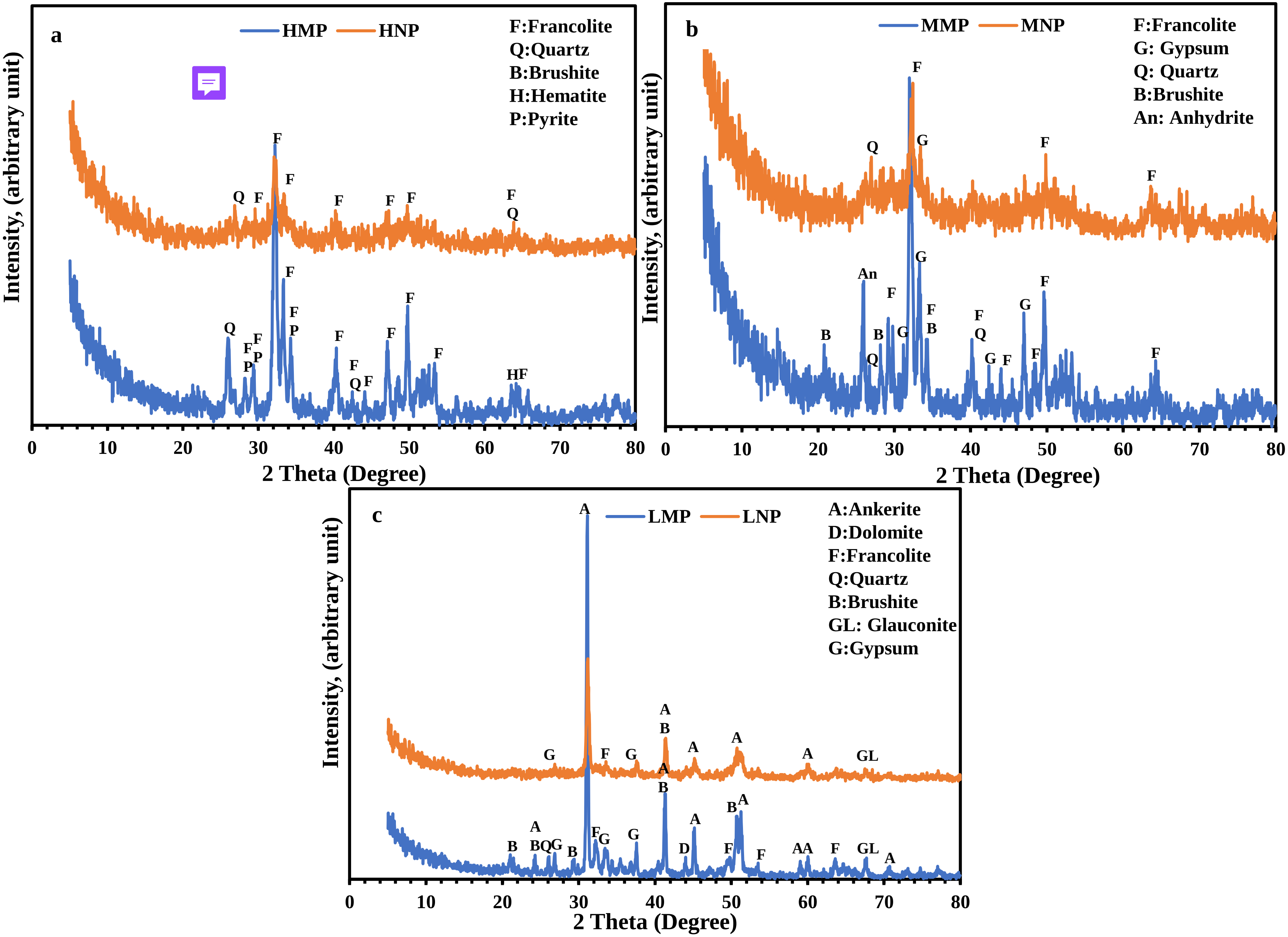
a
HMP
HNP
F:Francolite
Q:Quartz
B:Brushite
H:Hematite
P:Pyrite
Intensity, (arbitrary unit)
2 Theta (Degree)
b
MMP
MNP
F:Francolite
G: Gypsum
Q: Quartz
B:Brushite
An: Anhydrite
c
LMP
LNP
A:Ankerite
D:Dolomite
F:Francolite
Q:Quartz
B:Brushite
GL: Glauconite
G:Gypsum

Fig. 4

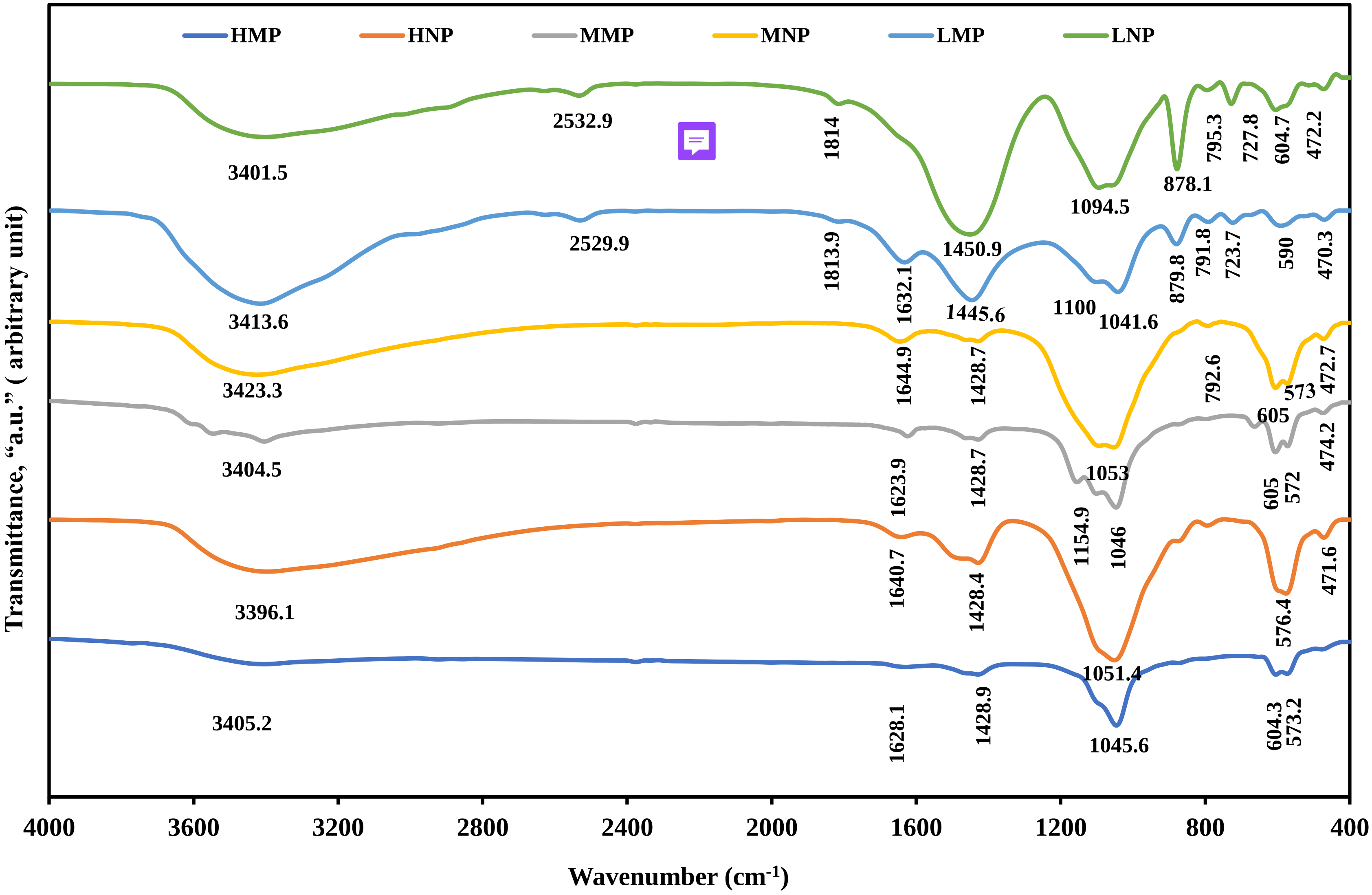
HMP
HNP
MMP
MNP
LMP
LNP
3401.5
2532.9
1814
1450.9
1094.5
878.1
795.3
727.8
604.7
472.2
3413.6
2529.9
1813.9
1632.1
1445.6
1100
1041.6
879.8
791.8
723.7
590
470.3
3423.3
1644.9
1428.7
792.6
605
573
472.7
3404.5
1623.9
1428.7
1053
1154.9
1046
605
572
474.2
3396.1
1640.7
1428.4
1051.4
576.4
471.6
3405.2
1628.1
1428.9
1045.6
604.3
573.2
Transmittance, "a.u." (arbitrary unit)
4000
3600
3200
2800
2400
2000
1600
1200
800
400
Wavenumber (cm-1)

Fig. 5

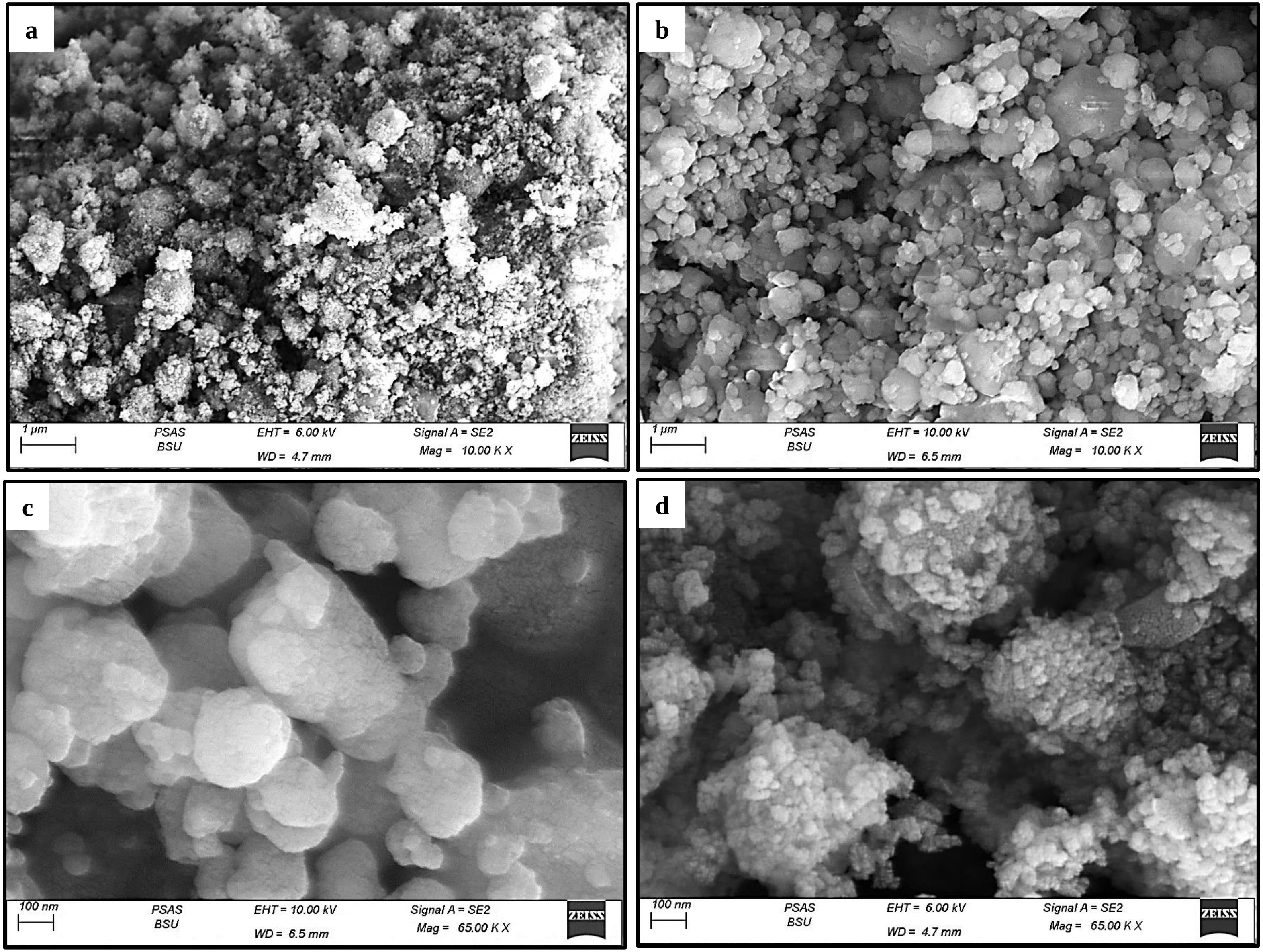
a
1 µm
PSAS
BSU
EHT = 6.00 kV
WD = 4.7 mm
Signal A = SE2
Mag = 10.00 K X
ZEISS
b
1 µm
PSAS
BSU
EHT = 10.00 kV
WD = 6.5 mm
Signal A = SE2
Mag = 10.00 K X
ZEISS
c
100 nm
PSAS
BSU
EHT = 10.00 kV
WD = 6.5 mm
Signal A = SE2
Mag = 65.00 K X
ZEISS
d
100 nm
PSAS
BSU
EHT = 6.00 kV
WD = 4.7 mm
Signal A = SE2
Mag = 65.00 K X
ZEISS

Fig. 6

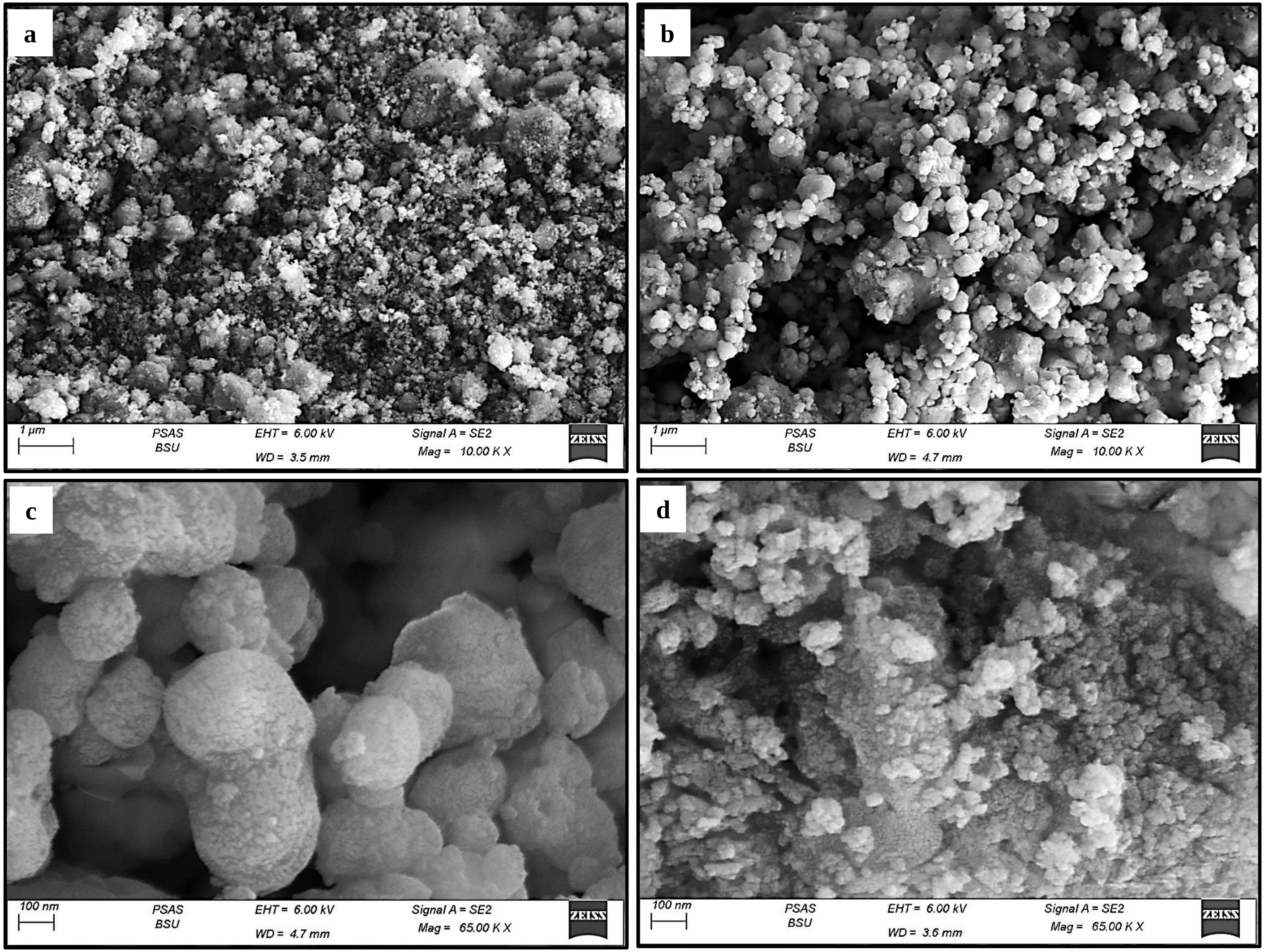
a
1 μm
PSAS
BSU
EHT = 6.00 kV
WD = 3.5 mm
Signal A = SE2
Mag = 10.00 K X
b
1 μm
PSAS
BSU
EHT = 6.00 kV
WD = 4.7 mm
Signal A = SE2
Mag = 10.00 K X
c
100 nm
PSAS
BSU
EHT = 6.00 kV
WD = 4.7 mm
Signal A = SE2
Mag = 65.00 K X
d
100 nm
PSAS
BSU
EHT = 6.00 kV
WD = 3.6 mm
Signal A = SE2
Mag = 65.00 K X

Fig. 7

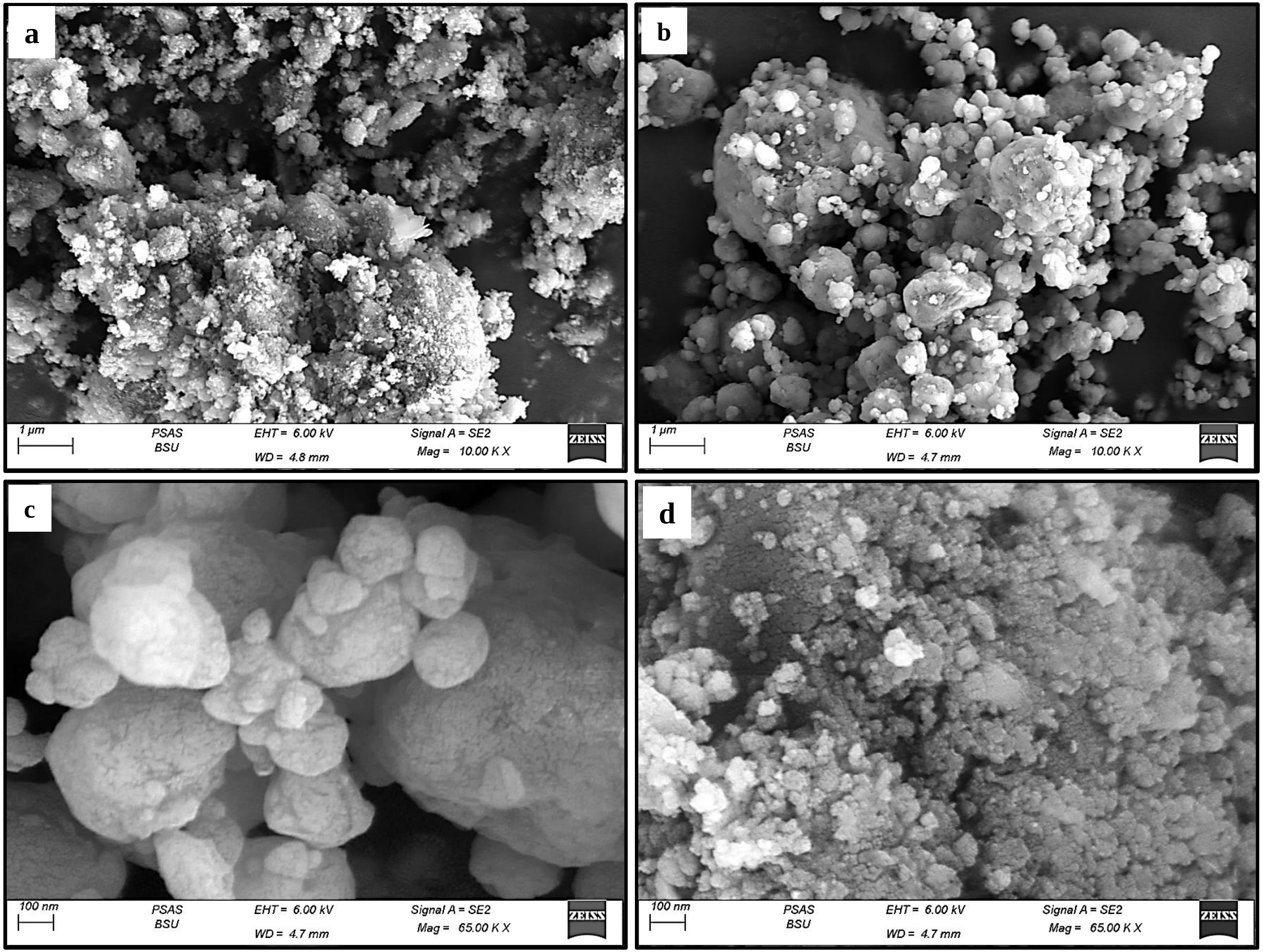
a
1 µm
PSAS
BSU
EHT = 6.00 kV
WD = 4.8 mm
Signal A = SE2
Mag = 10.00 K X
b
1 µm
PSAS
BSU
EHT = 6.00 kV
WD = 4.7 mm
Signal A = SE2
Mag = 10.00 K X
c
100 nm
PSAS
BSU
EHT = 6.00 kV
WD = 4.7 mm
Signal A = SE2
Mag = 65.00 K X
d
100 nm
PSAS
BSU
EHT = 6.00 kV
WD = 4.7 mm
Signal A = SE2
Mag = 65.00 K X

Fig. 8

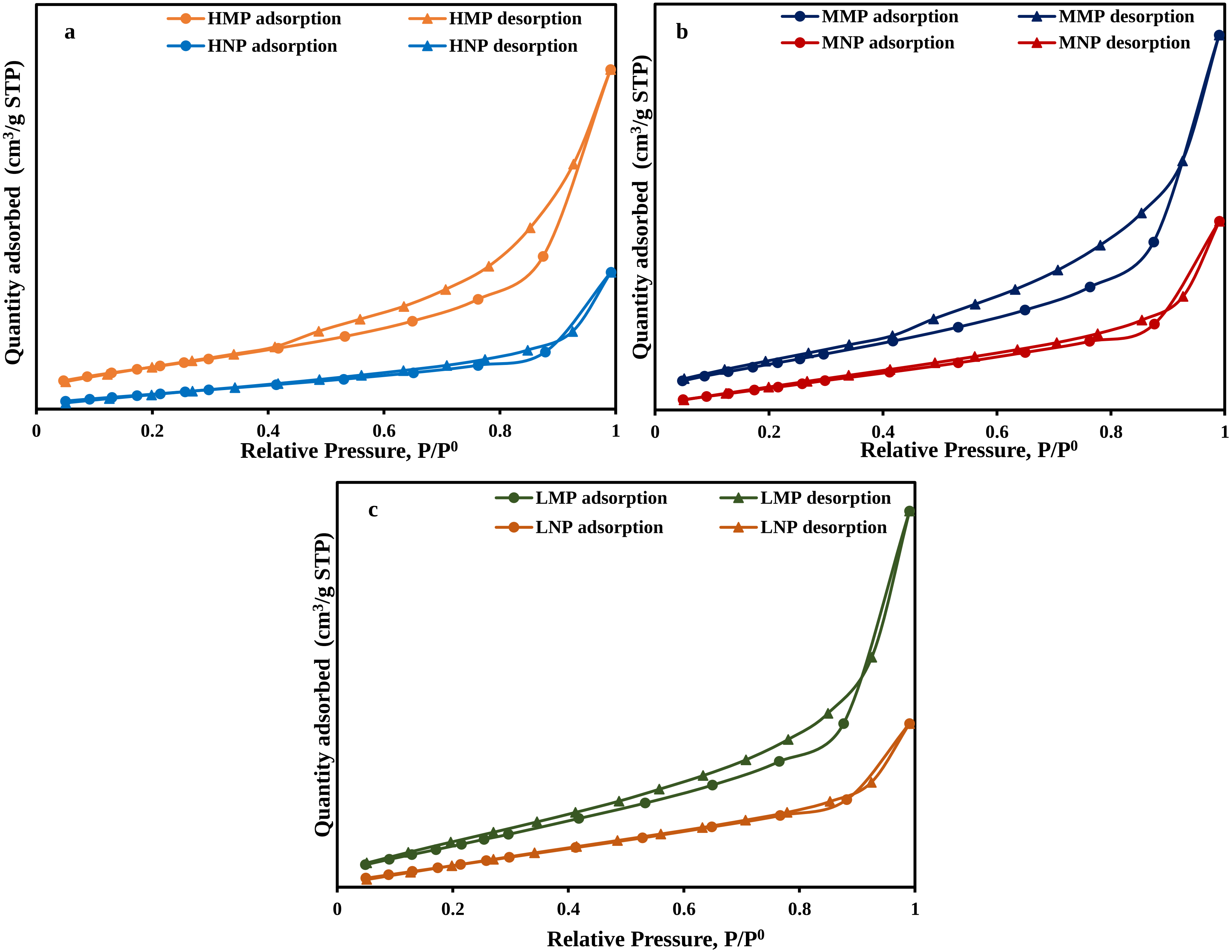

a
HMP adsorption
HMP desorption
HNP adsorption
HNP desorption
Quantity adsorbed (cm3/g STP)
Relative Pressure, P/P0
0
0.2
0.4
0.6
0.8
1
b
MMP adsorption
MMP desorption
MNP adsorption
MNP desorption
c
LMP adsorption
LMP desorption
LNP adsorption
LNP desorption

Fig. 9

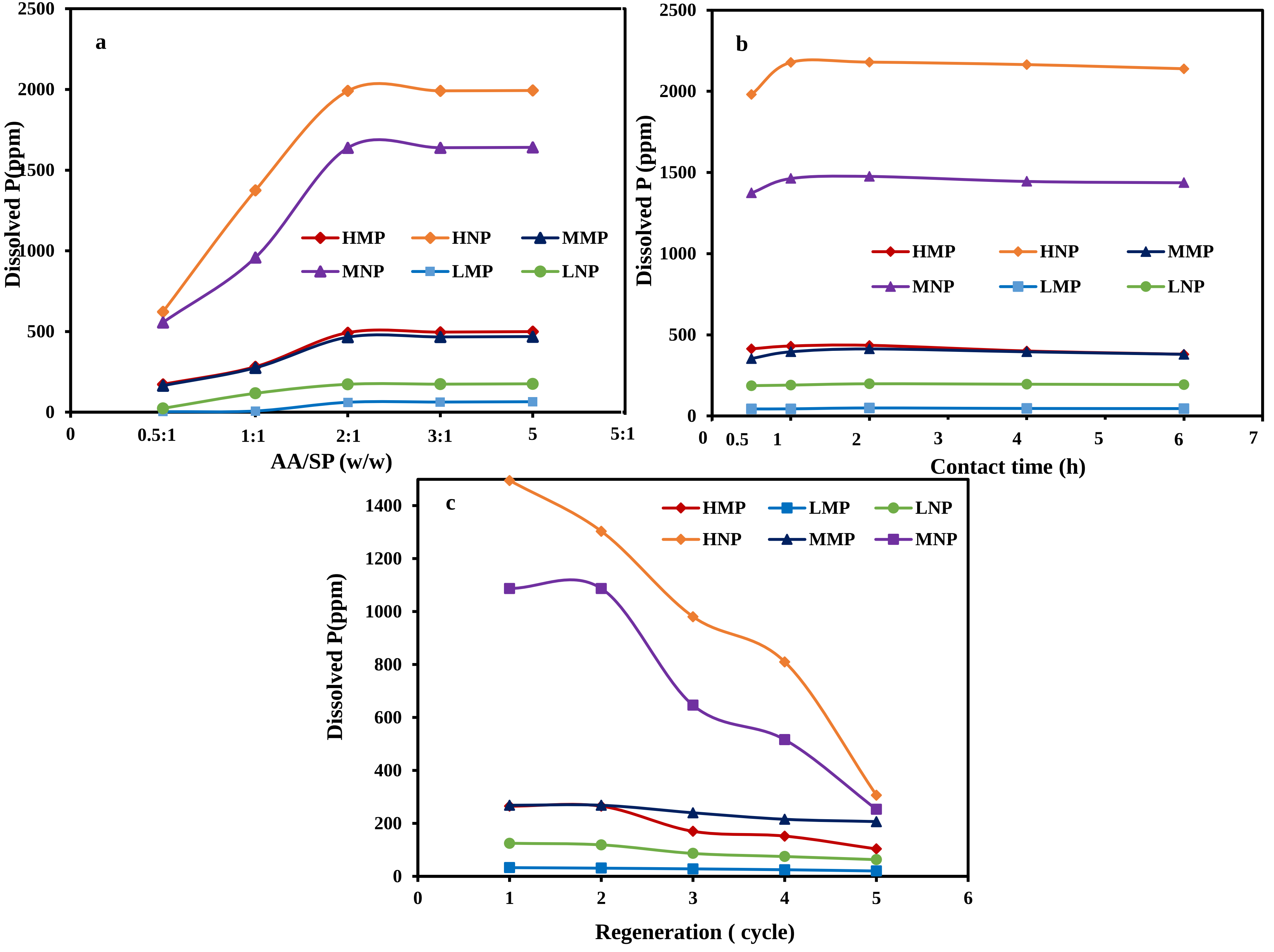

a
b
c
HMP
HNP
MMP
MNP
LMP
LNP
Dissolved P(ppm)
Dissolved P (ppm)
AA/SP (w/w)
Contact time (h)
Regeneration (cycle)
0
500
1000
1500
2000
2500
0.5:1
1:1
2:1
3:1
5
5:1
0.5
1
2
3
4
6
7
200
400
600
800
1200
1400

Fig. 10

Figure Click here to access/download;Figure;Figure captions.docx

Figure captions

Fig. 1. Field photographs showing the stratigraphic description of Duwi Formation: (a) Eastern part, (b) Western part of Liffyia - Maghrabi Sector in Abu Tartur mine.

Fig. 2. Petrographical images of: High-grade phosphate (HMP) in PPL (a, c & e) and XPL visions (b, d & f): Peloid grains (PL), Dolomite rhombs (D), Ooids (O), Fish bones (FB), Quartz (Q) and Iron oxide (F).

Fig.3. Petrographical images of: Medium-grade phosphate (MMP) in PPL (a), and low-grade phosphates in XPL visions (b & c): Peloid grains (PL), Evaporites (Eva), Dolomite rhombs (D), Quartz (Q) and Glauconite (G).

Fig. 4. X-ray diffraction pattern of the precursor micro-sizes in comparison with its modified nano-sized phosphates: HMP/ HNP (a), MMP/ MNP (b) and (c) LMP/LNP.

Fig. 5. FT-IR spectra of the precursor micro-sizes in comparison with its modified nano-sized phosphates of HMP, HNP, MMP, MNP, LMP, and LNP samples.

Fig. 6. SEM images of HMP (a & b) and HNP (c & d).

Fig. 7. SEM images of MMP (a & b) and MNP (c & d).

Fig. 8. SEM images of LMP (a & b) and LNP (c & d).

Fig. 9. N_2 adsorption-desorption isotherms of: HMP/HNP (a), MMP/MNP (b) and LMP/LNP (c).

Fig. 10. Effects of acetic acid concentration (a), reaction time (b) on P dissolution rate of both micro- and nanosized PR fractions. Regeneration studies display the magnitude of P dissolution rate along five leaching cycles of both micro- and nanosized PR fractions (c).
