# Supplementary material for: Nanoscale grinding: Unlocking the nutrient potential of oxidized phosphate rocks for sustainable fertilizer innovation
Source: PLoS One. 2025 May 9;20(5):e0321095. doi: 10.1371/journal.pone.0321095 (PMC12063831; doi:10.1371/journal.pone.0321095)
Supplement: Attachment — Submitted filename: response to reviwer comments.docx [file pone.0321095.s004.docx]

**Response for Reviewers' comments**

Firstly, the authors sincerely acknowledge the valuable comments and suggestions provided by the reviewers and the editor, which have significantly contributed to improving the clarity, scientific rigor, and overall quality of this manuscript. Their constructive feedback has helped refine our analyses, strengthen the discussion, and enhance the manuscript to meet the high scientific standards of *PLOS ONE journal*. We greatly appreciate their time and effort in evaluating our work.

**Reviewer #1**

**Comment:-**

I found the English in the manuscript well-written, although I am not a native English speaker. Nonetheless, some improvements are needed to enhance the manuscript’s quality.

**Response:-**

Authors appreciate the reviewer's positive feedback regarding the language quality of the manuscript. To further enhance clarity and readability, authors have carefully reviewed and refined the text, making necessary improvements in phrasing, grammar, and structure where needed. Authors believe these revisions have strengthened the overall quality of the manuscript and improved its readability."

**Comment:-**

The authors related the cominuation to the formation of amorphous and to change in the chemical composition. This topic needs be better explore with reference support. For example, I indicate the paper DOI: 10.1021/acsnano.9b05157 that shows the influence of grain size in the XRD data. The XRD does not indicate increases of amorphous phase in the samples with cominuation.
**Response:-**

Authors appreciate the reviewer's insightful comment and have carefully reviewed the suggested reference (DOI: 10.1021/acsnano.9b05157). Authors acknowledge that the XRD patterns do not indicate a significant increase in amorphous content but rather a subtle broadening of reflections, suggesting partial structural disorder rather than complete amorphization. To better support our discussion, authors have expanded this section with references on the influence of comminution on crystallinity and chemical composition changes. Studies have shown that mechanical grinding can induce lattice distortions, reduce crystallite size, and promote structural rearrangements, which can influence material reactivity and phase transitions [3]. To improve clarity, authors have revised the manuscript to better reflect these points and have cited additional references to support the discussion *(Please check the revised subsection “3.2. Characterization of Prepared Samples” XRD part and other parts related to this topic in the revised manuscript in red color).*

**Comment:-**

The manuscript need more support of reference in the discussion topic.

**Response:-**

Authors appreciate the reviewer's suggestion and have carefully revised the discussion section to incorporate additional references that support our findings. Relevant literature on specific topics, e.g., grinding effects on crystallinity, dissolution mechanisms, phosphate mineral transformations, were included, ensuring that the interpretations are well-supported by previous studies. These additions strengthen the scientific basis of discussion and align our results with existing research. The revised manuscript contains an expanded reference list to enhance credibility and context." *(Please check the revised manuscript and reference list).*

**Comment:-**

The nonconformity between chemical analyses of samples as well as FT-IR analysis in the different granulometry samples must be redone to check if it is not an analytical problem.

**Response:-**

Authors appreciate the reviewer's concern regarding the consistency of chemical and FT-IR analyses across different granulometry samples. To ensure accuracy, authors carefully verified the data by rechecking instrument calibration, baseline corrections, and measurement repeatability. The observed variations are consistent across multiple measurements and are likely attributed to real differences in chemical composition and structural modifications induced by grinding, rather than analytical errors. Additionally, the changes in FT-IR spectra align with expected transformations due to particle size reduction and mechanochemical effects. However, to further confirm the reliability of our data, authors have critically reassessed the results and ensured their consistency before finalizing the discussion.

**Comment:-**

I added some comments in the pdf file. The green marks indicate some doubt that need improved/clarified.

**Response:-**

Per your kind comments on the pdf file, authors would like to elaborate that each comment was considered carefully as illustrated below in this response file, as well as in the revised manuscript *(Please check the revised manuscript in red color).*

**Comment:-**

A location map, with regional indication and geology is mandatory.
**Response:-**

Authors appreciate the reviewer's comment and have now included a location map (Figure 1) providing regional context and geological background relevant to our study area. The map illustrates the study site, surrounding geological formations, and key lithological features to enhance spatial understanding. This addition aligns with standard geological reporting practices and improves the manuscript's clarity.

**Comment:-**

Put figures 2 and 3 in a single figure as well as figures 6 and 7. This will help to compare the samples characteristics. The other figures are very good.
**Response:-**

Done *(Please check the revised figures in the revised manuscript).*

**Comment:-**

Please do not use “&” as synonyms of “and”

**Response:-**

Done *(Please check the revised manuscript in red color).*

**Comment:-**

Although I indicated minor revisions, a very careful revision needs to be done by the authors to increase the quality of future publication.

**Response:-**

Authors sincerely appreciate the reviewer's constructive feedback and detailed suggestions, which have helped to improve the clarity and scientific rigor of this manuscript. Authors have carefully addressed all comments, revised key sections for better clarity, and ensured consistency across the data interpretation, discussions, and figures. Additionally, authors have refined the language and structure of the manuscript to enhance readability and overall quality. So, authors are confident that these revisions have strengthened the manuscript and thank the reviewer for his valuable insights."

**Comments on the pdf file**

**Comment:-**

In the abstract section the reviewer cited “*Reduction or lower?”* regarding the following cited sentence “*Despite the reduction in their P_2_O_5_ contents, HNP (31.23 wt.% and MNP (24.22 wt.%), astoundingly outperformed their micro-sized equivalents (HMP: 35.70 wt.%, MMP: 27.92 wt.%) in P dissolution*.”

**Response:-**

Thank you for your kind comment. The authors would like to illustrate that the use of word “reduction” is more illustrative than the use of “lower” word, because the P_2_O_5_ content in the HNP (31.23wt.%) & and MNP (24.22 wt.%) samples was actively decreased with grinding compared to the precursor samples (HMP: 35.70 wt.%, MMP: 27.92 wt.%).

**Comment:-**

In the introduction section the reviewer cited “*and/or increase in the amount of immobile P and its waste??*” regarding the following cited sentence in the manuscript “*Over time, excessive use of soluble P fertilizers can lead to soil saturation and reduced sorption capacity, potentially resulting in increased P losses [6].*”

**Response:-**

Per your kind inquiry about the cited sentence in the article “*and reduced sorption capacity, potentially resulting in increased P losses [6]*” the authors agree with the reviewer that the reduction in P sorption capacity can be ascribed to the increase in the amount of immobile P and hence its waste. Therefore, the suggested sentence was included in the revised article (*Please check the revised introduction section in red color*).

**Comment:-**

In the introduction section the reviewer cited “or form aluminium-phosphats that is insoluble” regarding the following cited sentence in the manuscript “*The soluble phosphorus that exceeds plant requirements is discharged into aquatic systems, resulting in algal blooms that can block sunlight and cause oxygen depletion,……….*”

**Response:-**

Per your kind suggestion the suggested sentence by the reviewer “*or form aluminum-phosphates that is insoluble”* was included in the text (*Please check the revised introduction section in red color*).

**Comment:-**

In the introduction section the reviewer cited “*this word is used only for sedimentary origin*” about the used “Phosphorites” word” regarding the following cited sentence in the manuscript “*Phosphorites, which are categorized into four types based on their origin:…….”*

**Response:-**

Thank you for your insightful observation. The authors agree with the reviewer comment, that phosphorites are used for phosphates of sedimentary origin, and to avoid confusion, phosphorite was replaced by phosphate resources ***(****Please check the revised introduction section in red color****).***

**Comment:-**

In the introduction section the reviewer cited “*Add references*” for the following sentence cited sentence in the manuscript “*biogenic deposits (bird and bat guano accumulations), are widely distributed in many parts of the world”.*

**Response:-**

Done ***(****Please check the revised introduction section in red color* *and the revised reference list****).***

**Comment:-**

In subsection “2.2. Preparation of samples” the reviewer cited “*Please use distilled water avoid this type of abbreviation*”. regarding the following sentence cited sentence in the manuscript “After washing several times with distilled water and drying in an oven drier at 70˚C overnight….”.

**Response:-**

Done ***(****Please check the revised subsection “2.2. Preparation of samples” and the other parts of the revised manuscript in red color****).***

**Comment:-**

In subsection “3.2. Characterization of prepared samples” the reviewer cited “*What is the mineralogical composition? indicate what it is*” regarding the mudclasts (peloids) in following cited sentence in the manuscript *“The detailed microscopic investigation revealed that the dominant composition of all the addressed PR grades are mudclasts (peloids)…….”*

**Response:-**

Thanks for your remarkable comment. Mudclasts (Peloids) refer to fragments of phosphatic mudstones, but sometimes contain silt-sized detrital grains such as quartz [1] *(Please check the revised subsection “3.2. Characterization of Prepared Samples” of the revised manuscript in red color).*

**Comment:-**

In subsection “3.2. Characterization of prepared samples” the reviewer cited “What is the mineralogical composition? indicate what it is” regarding the *phosphatic grains* in the following cited sentence in the manuscript *“On the other hand, the low-grade PRs are marked by high frequency of dolomite rhombohedra surrounding the phosphatic grains (Fig.3b-c).”*

**Response:**

Thanks for your remarkable comment. Phosphatic grains in this region are predominantly composed of minerals like fluorapatite (Ca₅(PO₄)₃F) and francolite (Ca₅(PO₄,CO₃)₃F)[2], but the most commonly encountered variant is francolite ***(****Please check the revised subsection “3.2. Characterization of Prepared Samples” in the revised manuscript in red color****).***

**Comment:-**

In subsection “3.2. Characterization of prepared samples”, XRF part, the reviewer cited “what ferrous components?” regarding the following cited sentence in the manuscript “*The increase in Fe_2_O_3_ content could be attributed to the oxidation of the ferrous components of their precursor micro-fractions with grinding*”

**Response:-**

Per your kind comment, the authors would like to illustrate that based on the mineralogical composition of the precursor phosphate rock samples, the most likely ferrous components could be **:-**

1. Glauconite (K, Na)(Fe³⁺,Fe²⁺,Mg, Al)₂(Si, Al)₄O₁₀(OH)₂ – A Fe²⁺- containing clay mineral found in phosphate deposits.
2. Ankerite (Ca (Fe²⁺, Mg, Mn) (CO₃)₂) – A carbonate mineral that can contain ferrous iron.
3. Pyrite (FeS₂) – While primarily Fe²⁺-S based, it can oxidize into Fe³⁺ phases like goethite or hematite upon exposure to air and moisture.

Therefore, to strengthen the previously introduced sentence in the manuscript, this sentence was modified as follow in the revised manuscript: *After intensive grinding to nanoscale, both Fe₂O₃ and LOI contents in HNP and MNP increased (Table 2). The rise in Fe₂O₃ content can be attributed to the oxidation of ferrous components, such as Fe²⁺-bearing minerals like glauconite, ankerite, and pyrite, which were present in the precursor micro-phosphate fractions before grinding* *(Please check the revised subsection “3.2. Characterization of Prepared Samples” in the revised manuscript in red color).*

**Comment:-**

In subsection “3.2. Characterization of prepared samples”, XRF part, the reviewer cited “this is not correct, please read DOI: 10.1021/acsnano.9b05157” regarding the following cited sentence in the manuscript “*the approximate destruction of the crystalline structure of both phosphatic and nonphosphatic components…..*”

**Response:-**

The authors appreciate the reviewer's insightful comment and have reviewed the suggested reference (DOI: 10.1021/acsnano.9b05157). Authors also acknowledge that the mechanochemical reduction in crystallinity (increases the amorphous nature of HNP and MNP) may influence structural interpretation, particularly in XRD analysis (i.e., concerning peak broadening and the quantification of amorphous content) [3]. While our results indicate increased LOI (Loss on Ignition) values, suggesting higher moisture retention. This suggests that the enhanced moisture uptake is more likely due to structural disorder. Therefore, the manuscript has been revised accordingly to clarify this as follow: *Similarly, the elevated LOI values suggest greater moisture retention, likely due to structural disorder and increased surface energy in both phosphatic and non-phosphatic components because of intensive grinding. However, interpreting the impact of crystalline structure disruption remains complex, particularly regarding peak broadening and the quantification of amorphous content (Please check the revised subsection “3.2. Characterization of Prepared Samples” in the revised manuscript in red color).*

**Comment:-**

In subsection “3.2. Characterization of prepared samples”, XRF part, the reviewer cited “*This is very unusual, are you sure about this? What is the technical explanation?”*, regarding the following cited sentence in the manuscript “*This could also be attributed to the approximate destruction of the francolite in these fractions (HNP & MNP) with higher intensity than that of LNP during the grinding process.*”

**Response:-**

Authors appreciate the reviewer's observation and have carefully re-evaluated the cited results. The reduction in P₂O₅ content in HNP and MNP can be attributed to the preferential breakdown of francolite (carbonate-fluorapatite) into smaller, more reactive fragments during intensive grinding. This process leads to an increased release of loosely bound phosphate species, which may have been lost due to surface interactions or volatilization during sample preparation. Additionally, grinding promotes structural disorder, which can alter the elemental distribution within the sample, affecting the XRF quantification of P₂O₅. On the other hand, the slight increase in P₂O₅ content in LNP may be due to the mechanical breakdown of gangue minerals (e.g., dolomite, ankerite, or silicates) surrounding phosphatic phases. This effect could expose more phosphate-rich regions that were previously shielded by non-phosphatic matrices, leading to a localized enrichment in P₂O₅. This interpretation aligns with our XRD and XRF findings, which suggest a more resistant mineralogical structure in LNP compared to HNP and MNP.

To strengthen this explanation, the manuscript has been revised to clarify this mechanism and its possible implications as follow: *Moreover, the P₂O₅ content decreased in HNP and MNP, whereas it slightly increased in LNP from 8.01 to 8.9 wt.% (Table 2). This reduction in HNP and MNP can be attributed to the preferential breakdown of francolite (carbonate-fluorapatite) into smaller, more reactive fragments during intensive grinding. This process may have led to the release of loosely bound phosphate species, which could have been lost due to surface interactions or volatilization. Additionally, grinding-induced structural disorder may have influenced the redistribution of elements, impacting XRF quantification. In contrast, the slight increase in P₂O₅ content in LNP could be due to the mechanical breakdown of gangue minerals (e.g., dolomite, ankerite, or silicates), which exposed more phosphate-rich regions that were previously embedded within the mineral matrix (Please check the revised subsection “3.2. Characterization of Prepared Samples” XRF part of the revised manuscript in red color).*

**Comment:-**

In subsection “3.2. Characterization of prepared samples”, XRF part the reviewer cited “*this is unbelievable. The analysis must be redone, it may just be a small reading error on the equipment. The only way there can be a decrease in the concentration of an element is if it is volatile*” regarding the following cited sentence in the manuscript “*Similarly, the CaO and F contents were slightly reduced after grinding, especially in the high and medium grade fractions of nano-size***”**

**Response:-**

Authors appreciate the reviewer's concern and have carefully reconsidered these findings. The observed slight decrease in CaO and F contents in the high- and medium-grade nano-sized fractions is unlikely due to volatilization, as neither Ca nor F are volatile under the grinding conditions used in this study. Instead, this decrease can be attributed to the mechanical breakdown of carbonate-fluorapatite (francolite) in both HNP, which may have led to a redistribution of Ca and F within the sample matrix rather than an actual loss. As well, the decomposition of Ca-Sulfate Phases (Gypsum and Anhydrite in MNP) may have affected the hydration state of gypsum (CaSO₄·2H₂O), leading to partial transformation into anhydrite (CaSO₄), which could impact XRF detection of CaO. Additionally, grinding-induced amorphization and particle agglomeration could have influenced XRF detection efficiency, affecting the apparent concentrations. Changes in particle packing and sample homogeneity during pellet preparation may have also contributed to variations in the detected values.

To ensure the reliability of our results, the XRF calibration was rechecked, confirming that the trend is consistent across multiple measurements. However, authors acknowledge that further validation using complementary techniques such as ICP-OES or EDS could provide additional confirmation and will be considered in future studies. Therefore, the manuscript has been revised to clarify this explanation *(Please check the revised subsection “3.2. Characterization of Prepared Samples” XRF part the revised manuscript in red color).*

**Comment:-**

In subsection “3.2. Characterization of prepared samples”, XRD part, the reviewer cited “*see comment above”,* regarding the following cited sentence in the manuscript “……*reflects the approximately mild impact of grinding on the crystallinity of LMP (Fig. 4c). Such fragile impact could be attributed to the high LOI content (> 32 wt. %) in the LMP compared to the other investigated grades as displayed by XRF data. This high ratio complicates the grinding process to nano-size”.*

**Response:-**

authors appreciate the reviewer's comment and have revised the discussion to ensure consistency with the XRD observations. The statement regarding the ‘mild impact’ of grinding on LMP crystallinity has been adjusted to better reflect the data. As seen in Figure 4c, the XRD patterns indicate only a slight broadening of reflections, suggesting that while some structural disorder occurs, the crystallinity of LMP remains relatively high. The high LOI content (>32 wt.%) in LMP likely contributed to this behavior by complicating the grinding process, potentially promoting particle agglomeration and reducing overall grinding efficiency. This aligns with observations that materials with high volatile content tend to be more resistant to size reduction. To improve clarity, authors have refined the manuscript text accordingly *(Please check the revised subsection “3.2. Characterization of Prepared Samples” XRD part the revised manuscript in red color).*

**Comment:-**

In subsection “3.2. Characterization of prepared samples”, FT-IR part, the reviewer cited “*need reference support*” regarding the following cited sentence in the manuscript “*the release of these volatiles in an approximately closed grinding system has triggered pressure building up, justifying the remerging of these vapors in H_2_CO_3_ form*.”

**Response:-**

Done, reference [4] was already added ***(****Please check the revised subsection “3.2. Characterization of Prepared Samples” FT-IR part of the revised manuscript in red color).*

**Comment:-**

In subsection “3.2. Characterization of prepared samples”, FT-IR part, the reviewer highlighted the following sentence in the manuscript “*Similarly, the strength of Si-O and (PO4)3 - groups in the nano-size fractions could be attributed to the gained degree of amorphization that was induced by intensive grinding*”

**Response:-**

Done ***(****Please check the revised subsection “3.2. Characterization of Prepared Samples” FT-IR part of the revised manuscript in red color).*

**Comment**

In subsection “3.2. Characterization of prepared samples”, SEM part, the reviewer typed “*????*” as a comment for the following sentence in the manuscript *“………..difference in durability* ……..”

**Response:-**

Per your kind inquiry and to avoid confusion, the durability word was replaced by toughness to avoid any confusion *(Please check the revised subsection “3.2. Characterization of Prepared Samples” SEM part of the revised manuscript in red color).*

**Comment:-**

In subsection “3.2. Characterization of prepared samples”, SEM part, the reviewer cited “*LOI can change* *in consequence of heat during jaw crushing and mill*” regarding the following cited sentence in the manuscript “ *…… the LOI contents among these fractions…….*”

**Response:-**

The authors 100% agree with the reviewer’s citation that LOI can change in consequence of heat during milling process *(Please check the revised subsection “3.2. Characterization of Prepared Samples” SEM part of the revised manuscript in red color).*

**Comment:-**

In subsection “3.2. Characterization of prepared samples”, SEM part, the reviewer cited “*reference support*” regarding the following cited sentence in the manuscript “*The spheroidal nature of the produced particles was inversely proportional with the LOI………….”*

**Response:-**

Per the reviewer’s request, the authors acknowledge the need for reference support for the statement: *'The spheroidal nature of the produced particles was inversely proportional to the LOI*.' However, as direct literature explicitly linking particle sphericity to LOI is unavailable, the authors have decided to remove this statement to avoid any potential confusion (*Please check the revised subsection “3.2. Characterization of Prepared Samples” SEM part of the revised manuscript).*

**Comment:-**

In subsection “3.3 Experiments” the reviewer highlighted “*Experiments*” as a title for the subsection.

**Response:-**

Per your kind inquiry the subsection title was changed into ‘*leaching experiments*’ to avoid any confusions *(Please check the revised subsection “3.3. Leaching Experiments” in the revised manuscript in red color).*

**Comment:-**

In subsection “3.3.2 Effect of Reaction Time” the reviewer cited “*the leaching data indicate this*” regarding the following cited sentence in the manuscript “*2h was selected* *as an equilibrium time*”.

**Response:-**

Authors appreciate the reviewer's comment and understand the need for clarification. The selection of 2 hours as the equilibrium time was based on leaching data trends, which indicated that the contribution of H⁺ protons of the employed acetic acid did not change significantly beyond this point. Specifically, the P dissolution rate showed insignificant improvement for both micro/nano size PR fractions after 2 hours, suggesting that the reaction had reached equilibrium. This trend can be observed in Fig.(8b), where the dissolution rate of P plateaued, indicating minimal further interaction with H⁺ ions.

**Comment:-**

In subsection “3.3.2 Effect of Reaction Time” the reviewer cited “*I can´t understand this*” regarding the following cited sentence in the manuscript “*with some preference of the former (HMP = 380- 431 ppm)*”.

**Response:-**

Authors appreciate the reviewer's comment and have revised the sentence for clarity. The term ‘*preference’* was intended to indicate that HMP exhibited slightly higher P dissolution values compared to MMP. To avoid ambiguity, authors have reworded the sentence to explicitly state that HMP showed a dissolution range of 380–431 ppm, which is slightly higher than MMP. The revised text ensures that the comparison between the two micro-fractions is clearer *(Please check the revised subsection “3.3.2 Effect of Reaction Time” in the revised manuscript in red color).*

**Comment:-**

In subsection “3.3.2 Effect of Reaction Time” the reviewer cited “*high or low*?” regarding the following cited sentence in the manuscript “*the crystallinity nature of both LMP and LNP*

*samples*”

**Response:-**

Authors appreciate the reviewer's comment and have revised the sentence for clarity. The intended meaning was that LMP exhibits high crystallinity, while LNP has low crystallinity, and this structural difference, along with their low P₂O₅ content, contributes to their low P dissolution rates. The revised text explicitly distinguishes between the two samples to avoid any ambiguity *(Please check the revised subsection “3.3.2 Effect of Reaction Time” in the revised manuscript in red color).*

**Comment:-**

In subsection “3.3.3 Regeneration Studies” the reviewer cited “*what do you mean by regeneration studies?*” for the title of this subsection.

**Response:-**

Authors appreciate the reviewer's comment. In this study, "*regeneration studies*" refer to the assessment of the reusability of the PR materials after the P dissolution process. Specifically, authors evaluated whether the PR residues could be reutilized for further leaching reactions for multiple P dissolution cycles. To ensure clarity, authors have revised the subsection title to "Reusability Studies" to better reflect the content *(Please check the revised subsection “3.3.3 Reusability Studies” in the revised manuscript in red color).*

**Comment:-**

In subsection “3.3.3 Regeneration Studies” the reviewer cited “*what fraction?*” regarding the following sentence in the manuscript “*……fractions’ particles……”*

**Response:-**

Thank you for your comment. authors have clarified the term "*fractions' particles*" in the manuscript to specify the micro/nanoscale PR fractions (HMP, HNP, MMP, MNP, LMP, and LNP). So, for better clarity and to avoid any confusion or ambiguity, the sentence was modified as follow:” *This was probably assigned to the high concentration of particles from each of these micro/nanoscales PR fractions within a fixed volume of acetic acid that diminished the solid-liquid interface due to particles agglomeration/stacking, aligning with SEM data(Please check the revised subsection “3.3.3 Reusability Studies” in the revised manuscript in red color).*

**Comment:-**

On XRD figure, the reviewer cited “*The XRD data do not indicate increased in amorphicity with lower size grain, there is only a subtle slight broad reflection increase*”

**Response:-**

Authors appreciate the reviewer's comment and acknowledge that the increase in amorphicity with decreasing grain size is subtle rather than drastic. The observed broadening of XRD reflections, though slight, suggests a reduction in crystallite size and increased structural disorder, which are common effects of intensive grinding [3]. However, authors agree that the data does not indicate complete amorphization but rather a gradual transition toward a more disordered structure. To clarify this, authors have revised the XRD discussion to reflect that the increase in amorphicity is moderate and inferred from the observed peak broadening rather than a complete loss of crystallinity (*Please check the revised subsection “3.2. Characterization of Prepared Samples” XRD part of the revised manuscript in red color).*

**Comment:-**

On FT-IR figure, the reviewer cited “These analyses must be repeated to verify that there is no analytical problem”.

**Response:-**

. Authors appreciate the reviewer's concern regarding the FT-IR analyses. The spectra were collected using a well-calibrated instrument, and multiple measurements were performed to ensure data consistency. The obtained spectra were reproducible, and no analytical errors were detected. Additionally, standard baseline corrections and instrumental background subtraction were applied to minimize any potential artifacts.

To further confirm the reliability of the results, authors have carefully rechecked the FT-IR data, and the observed spectral features remain consistent with the expected functional group assignments. Therefore, authors believe the current data are accurate and representative.

**References**

1.	Baioumy H, Tada R. Origin of Late Cretaceous phosphorites in Egypt. Cretaceous Research. 2005;26(2):261-75. doi: <https://doi.org/10.1016/j.cretres.2004.12.004>.

2.	Roshdy O, Haggag E, Masoud A, Bertau M, Haneklaus N, Pavón S, et al. Leaching of rare earths from Abu Tartur (Egypt) phosphate rock with phosphoric acid. Journal of Material Cycles and Waste Management. 2022;25. doi: 10.1007/s10163-022-01558-8.

3. Cameron F. Holder and Raymond E. Schaak. Tutorial on Powder X‑ray Diffraction for Characterizing Nanoscale Materials. ACS Nano 2019, 13, 7359−7365. DOI: 10.1021/acsnano.9b05157

4. Hongbo Wang, Janek Zeuschner, Mikhail Eremets, Ivan Troyan & Jonathan Willams (2016). Stable solid and aqueous H_2_CO_3_ from CO_2_ and H_2_O at high pressure and high temperature. scientificreports 6:19902, DOI: 10.1038/srep19902
